# Intelligent driving intelligence test for autonomous vehicles with naturalistic and adversarial environment

**DOI:** 10.1038/s41467-021-21007-8

**Published:** 2021-02-02

**Authors:** Shuo Feng, Xintao Yan, Haowei Sun, Yiheng Feng, Henry X. Liu

**Affiliations:** 1 Department of Civil and Environmental Engineering, University of Michigan, Ann Arbor, MI USA; 2 University of Michigan Transportation Research Institute, Ann Arbor, MI USA

**Keywords:** Civil engineering, Mechanical engineering, Computer science

## Abstract

Driving intelligence tests are critical to the development and deployment of autonomous vehicles. The prevailing approach tests autonomous vehicles in life-like simulations of the naturalistic driving environment. However, due to the high dimensionality of the environment and the rareness of safety-critical events, hundreds of millions of miles would be required to demonstrate the safety performance of autonomous vehicles, which is severely inefficient. We discover that sparse but adversarial adjustments to the naturalistic driving environment, resulting in the naturalistic and adversarial driving environment, can significantly reduce the required test miles without loss of evaluation unbiasedness. By training the background vehicles to learn when to execute what adversarial maneuver, the proposed environment becomes an intelligent environment for driving intelligence testing. We demonstrate the effectiveness of the proposed environment in a highway-driving simulation. Comparing with the naturalistic driving environment, the proposed environment can accelerate the evaluation process by multiple orders of magnitude.

## Abstract (web summary)

Tests for autonomous vehicles are usually made in the naturalistic driving environment where safety-critical scenarios are rare. Feng et al. propose a testing approach combining naturalistic and adversarial environment which allows to accelerate testing process and detect dangerous driving events.

## Introduction

Autonomous vehicles (AVs) have attracted significant attention in recent years because of their potential to revolutionize transportation safety and mobility. One critical step in the development and deployment of AVs is to test and evaluate their driving intelligence, which indicates whether an AV can operate safely and efficiently without human intervention. However, current testing procedures for human-driven vehicles, such as Federal Motor Vehicle Safety Standards (FMVSS)^1^ and ISO 26262, only regulate automobile safety-related components, systems, and design features, without consideration of driving intelligence in completing driving tasks. To the best of the authors’ knowledge, to date there are no consensus nor standard procedures on how to test and evaluate AVs. During the past few years, although the problem of AV testing has been investigated extensively by various AV developers, government agencies, professional organizations, as well as academic institutions, the theory and methods to support such testing and evaluation are lacking^2,3^.

**Fig. 1:**
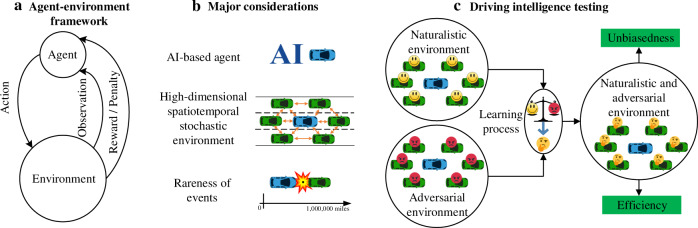
Driving intelligence testing with NADE. **a** Agent-environment framework. **b** Major challenges for agent-environment framework include the difficulty for applying traditional software validation methods for testing artificial intelligence (AI) based agents, the “curse of dimensionality” for modeling complex dynamic driving environment, and rareness of events of interest for driving intelligence testing. The blue vehicle denotes the autonomous vehicle, and the green vehicles denote background vehicles. **c** The NADE learns to balance the naturalistic environment and adversarial environment for driving intelligence testing of AVs based on the agent-environment framework, while ensuring unbiasedness and improving efficiency. The images of vehicles, balance scale, and explosion are previously published under the Creative Common CC0 1.0 Universal Public Domain Dedication. The image of smiley face is previously published under the Creative Commons Attribution-Share Alike 3.0 Unported license. The images of angry face and thinking face are previously published under the Creative Commons Attribution 4.0 International license. These images can be found from the followings links: https://commons.wikimedia.org/wiki/File:C3top.png; https://commons.wikimedia.org/wiki/File:Balanced_scale_of_Justice.svg; https://commons.wikimedia.org/wiki/File:Explosion-155624_icon.svg; https://commons.wikimedia.org/wiki/File:Mr._Smiley_Face.svg; https://commons.wikimedia.org/wiki/File:Twemoji12_1f621.svg; https://commons.wikimedia.org/wiki/File:Twemoji2_1f914.svg.

As shown in Fig. 1a, the prevailing state-of-the-art approach for AV testing uses the agent-environment framework^4^, through a combination of software simulation, closed-track testing, and on-road testing. The basic philosophy is to test the agents of AVs in a realistic driving environment, observe their performance, and make statistical comparisons to human driver performance. The challenge for AV testing, however, comes from three different aspects shown in Fig. 1b: First, the driving agent in AV is commonly developed based on statistics or artificial intelligence (AI) algorithms. The AI-based agent, which is usually a black box to external users, limits the use of traditional logic-based software verification and validation techniques^5^. Second, the driving environment is usually complex and stochastic. To represent the full complexity and variability of the environment, variables that define the environment are high dimensional, which can cause the “curse of dimensionality”. The stochasticity of the environment can also fail the traditional formal methods for absolute safety. Third, events of interest (e.g., accidents) for the driving intelligence test rarely happen, and the rareness of events can lead to the intolerable inefficiency issue for testing. Therefore, how to construct an intelligent testing environment that can test AV driving intelligence accurately and efficiently, with consideration of high dimensionality and the rareness of events, becomes the key to the AV testing problem.

Most existing methods use the naturalistic driving environment (NDE) for driving intelligence testing of AVs. For example, on-road methods test AVs in the real-world NDE, while most simulation methods test high-fidelity AV models in life-like simulations of NDE, such as Intel’s CARLA^6^, Microsoft’s AirSim^7^, NVIDIA’s Drive Constellation^8^, Google/Waymo’s CarCraft^9^, Baidu’s AADS^10^, etc. However, all these methods suffer from inefficiency issue, because of the “curse of dimensionality” and the rareness of events in NDE, as discussed above. It has been argued that hundreds of millions of miles and sometimes hundreds of billions of miles would be required to demonstrate the safety performance of AVs at the level of human-driven vehicles^11^. Not to mention that a brand-new testing process may be required if configurations of AVs are changed. It is inefficient even under aggressive simulation schemes. In fact, Waymo has only simulated 15 billion miles in total over the years, which is the world’s longest simulation test. To a certain extent, this inefficiency issue has hindered the progress of the AV development and deployment.

Towards solving the inefficiency issue, scenario-based approaches have been proposed. Based on the importance sampling (IS) theory, critical scenarios can be purposely designed for accelerating the efficiency of AV evaluation^12–17^. However, existing scenario generation methods can only be applied for scenarios that involve simple maneuvers of a very limited number of vehicles with very short duration, for instance, a cut-in maneuver from a background vehicle for a few seconds. They are far from representing the full complexity and variability of the real-world driving environment. For example, an AV driving in a highway-driving environment can involve various maneuvers (e.g., lane-changing, car-following, over-taking, etc.) of hundreds of vehicles for hours of time duration. Such a driving environment contains numerous distinctive spatiotemporal combinations of scenarios, which cannot be handled by existing scenario-based approaches.

Our approach to the construction of a simulation or test-track based AV testing environment has the following three contributions: First, our approach generates the driving environment that provides spatiotemporally continuous testing scenarios for AVs. Suppose you want to test an AV in an urban environment, our approach can drive the AV continuously for miles in the environment during one test, interacting with multiple background vehicles and experiencing different adversarial scenarios. Second, the generated environment provides statistically accurate testing results. Our approach ensures that the testing results (such as accident rates of different accident types) of AVs in the generated environment are unbiased with the NDE. Third, the generated environment addresses the inefficiency issue of the NDE. Comparing with the NDE, our approach reduces the testing time with multiple orders of magnitude for the same evaluation accuracy.

To achieve evaluation efficiency without loss of accuracy, our approach is based on NDE, but with sparse but intelligent adjustments. The resulting driving environment is both naturalistic and adversarial, in that most of the background vehicles (more generally, road users) follow naturalistic behaviors for most of the time, and only at selected moments, selected vehicles execute specific designed adversarial moves. As shown in Fig. 1c, the key to creating the naturalistic and adversarial driving environment (NADE) is to train the background vehicles in the NDE to learn when to execute what adversarial maneuver while ensuring unbiasedness and improving efficiency. The learning process is guided by our theoretical discovery below.

In essence, AV driving intelligence testing can be considered as a rare event estimation problem with high-dimensional variables. However, few existing methods can handle both the challenges of the rareness of events and high dimensionality. Testing AVs in NDE is an application of the Crude Monte Carlo (CMC) theory^18^, which suffers from inefficiency problem for rare events. The IS theory has been developed for solving the challenge of rare events, but it can only be applied in low-dimensional situations^19^. It was proved that its efficiency would decrease exponentially with the increase of dimensionality. Therefore, both CMC and IS have limitations for the rare event estimation problem with high-dimensional variables. However, people have not paid much attention to the advantage of the CMC theory for high dimensionality. We discover that, if there exists a small subset of variables that are critical to the rare events, applying IS theory with the small subset of variables while applying the CMC theory with the remaining variables can help overcome both the challenges of the rareness of events and high dimensionality. We provide a theoretical proof of this in Theorem 1 in Methods. This is significant as this can apply to a general set of problems with such characteristics. For safety-critical performance tests of AVs, fortunately, these small but critical variables exist because most of the vehicle accidents involve only a small number of vehicles in a short period^20^. According to the Fatality Analysis Reporting System (FARS), about 91.5% of fatal injuries suffered in motor vehicle traffic crashes in the United States in 2018 involved only one or two vehicles^21^.

As the construction of NADE is based on NDE, we propose a data-driven approach to resemble naturalistic behavioral patterns of background vehicles for the generation of NDE. The basic idea is to model NDE with the Markov decision process, calculate naturalistic distributions of vehicle maneuvers from naturalistic driving data, and sample vehicle maneuvers from the distributions. The NDE provides the foundation and benchmark for the generation and evaluation of NADE. To identify the small but critical variables for the generation of NADE, we propose a reinforcement learning approach to learn the challenge of background vehicle maneuvers to the AV under test. This is similar to the value network approach in AlphaGo^22^ as the maneuver challenges of background vehicles at any moment are interdependent with the AV maneuvers in the following time steps. In addition, as the specifics of the behavior model of the AV under test are usually unknown, we propose utilizing surrogate models (SMs) during the learning process. The construction of SMs provides an elegant way to leverage prior knowledge such as testing results for previous AV models. Based on the maneuver challenge, the principal other vehicles (POVs) can be identified from all surrounding background vehicles, and their maneuvers can be adjusted at critical moments. In such a manner, only the distributions of a small but critical set of variables are twisted according to the IS theory, while the remaining variables follow their naturalistic distributions. Such sparse but intelligent adjustment of NDE results in NADE.

We demonstrated the effectiveness of our method for AV testing in a highway driving environment based on a high fidelity simulation platform, CARLA^6^, and a highway traffic simulator^23^, though our method is also applicable for other driving environments, such as city driving. We utilized the naturalistic driving data (NDD) from the Safety Pilot Model Deployment (SPMD) program^24^ and the Integrated Vehicle-Based Safety System (IVBSS)^25^ at the University of Michigan, Ann Arbor. To validate the generated NADE, we constructed two representative AV agents based on driving behavior models and deep reinforcement learning techniques, respectively. The accident rates of the AVs were utilized for the driving intelligence measurement. We tested the AVs in NDE and NADE, respectively. Simulation results show that the NADE could significantly accelerate the evaluation process by multiple orders of magnitude with the same accuracy, comparing with the NDE-based method.

## Results

### Generation and evaluation of NDE

Generation of NDE is a prerequisite for unbiased simulation-based intelligence tests of AVs. It usually has two pillars. The first is creating realistic inputs to AVs’ sensors, such as photorealistic images that resemble real-world renderings. There exists a large body of literature on this topic based on computer graphics, physics-based modeling, robot motion planning, and augmentation techniques. In this paper, we achieved real-world renderings by using the open-source platform CARLA. The second is creating naturalistic behavioral patterns of traffic participants. Although human driving behaviors have been extensively investigated in the transportation engineering domain, most existing models were developed for traffic flow analysis purposes, which may not be suitable for driving safety assessment. To estimate AV’s safety performance, the probabilistic distributions of human driving behaviors at different driving conditions are critical. Only with naturalistic probabilistic distributions, simulation results can predict their performances in the real world. Therefore, the goal of NDE is to generate stochastic human driving behaviors, whose probabilistic distributions are consistent with the NDD.

In this paper, we present a simple yet effective data-driven approach to resemble the naturalistic behavioral distributions of vehicles. The basic idea is to model NDE with Markov decision process (MDP), calculate empirical distributions of vehicle maneuvers given vehicle states from NDD, and then sample vehicle maneuvers from the distributions. The decision process of vehicle maneuvers in NDE can be represented by a decision tree^15^. Each node of the tree denotes a specific realization of vehicle states, while each path denotes a specific realization of vehicle maneuvers. If all vehicles select their maneuvers by sampling from the naturalistic distributions, the driving environment results in NDE. The proposed method for NDE generation can be further improved by advanced data processing techniques^26–28^ and modeling techniques^29^, but we leave those for future studies.

**Fig. 2:**
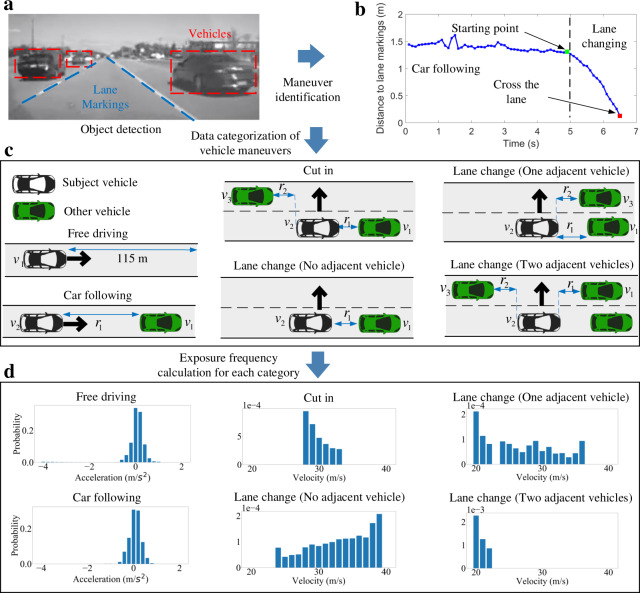
Data processing of the NDD. **a** Object detection of vehicles and lane markings for an example frame captured by cameras. **b** Identification of lane changing maneuvers by analyzing lateral distance to lane markings. **c** Data categorization of vehicle maneuvers considering surrounding vehicles. The image of vehicles is previously published at https://commons.wikimedia.org/wiki/File:C3top.png under the Creative Common CC0 1.0 Universal Public Domain Dedication. **d** Examples of empirical distributions of vehicle maneuvers for each category. States of the examples are *r*_1_ = 30 m *v*_1_ = *v*_2_ = 30 m s^−1^ (car following); *r*_1_ = 40 m, *r*_2_ = 21 m, *v*_1_ = *v*_2_ = *v*_3_ (cut in); *r*_1_ = 31 m, *v*_1_ = *v*_2_ (lane change, no adjacent vehicle); *r*_1_ = 20 m, *r*_2_ = 32 m *v*_1_ = *v*_2_ = *v*_3_ (lane change, one adjacent vehicle); and *r*_1_ = 28 m, *r*_2_ = 26 m, *v*_1_ = *v*_2_ = *v*_3_ (lane change, two adjacent vehicles).

To obtain naturalistic distributions, we collected NDD from the SPMD program and IVBSS at the University of Michigan, Ann Arbor. The SPMD database is one of the largest databases in the world that recorded naturalistic driving behaviors over 34.9 million travel miles from 2842 equipped vehicles in Ann Arbor, Michigan. In the database, there are 98 sedans equipped with the data acquisition system (DAS). In the IVBSS project, 108 randomly sampled drivers used sixteen Honda Accord vehicles with the DAS for over 40 days. Figure 2a shows an example frame captured by the Mobileye camera of the DAS equipped vehicles. At a frequency of 10 Hz, the data contain positions, speeds, and accelerations of all recorded vehicles, and measured both longitudinal and lateral distances between vehicles and lane markings. We queried the data with the following criteria: (1) vehicle was traveling on a highway; (2) vehicle was traveling at a speed between 20 m s^−1^ and 40 m s^−1^; (3) dry surface condition; (4) daylight condition. The resulting dataset represented more than 1.86 × 10^8^ points of data. By analyzing the lateral distance to lane markings, we identified a total number of 1.4 × 10^4^ lane-changing maneuvers (Fig. 2b). Considering the driving environment of the subject vehicle (SV), we further categorized the queried data into six groups: free driving, car following, cut in, lane change with zero, one, and two adjacent vehicles (Fig. 2c). The vehicle maneuvers were discretized into 33 actions: left lane change, 31 discrete longitudinal accelerations ([−4, 2] with 0.2 m s^−2^ discrete resolution), and right lane change. To simplify the maneuvers, longitudinal accelerations were assumed zero during the lane changing process. Then, the empirical distribution of each maneuver at each state was calculated by its exposure frequency in the dataset of the corresponding category. Figure 2d shows examples of the obtained distributions such as accelerations of the free driving and car following categories, and lane changing probabilities of the other four categories, given specific states.

**Fig. 3:**
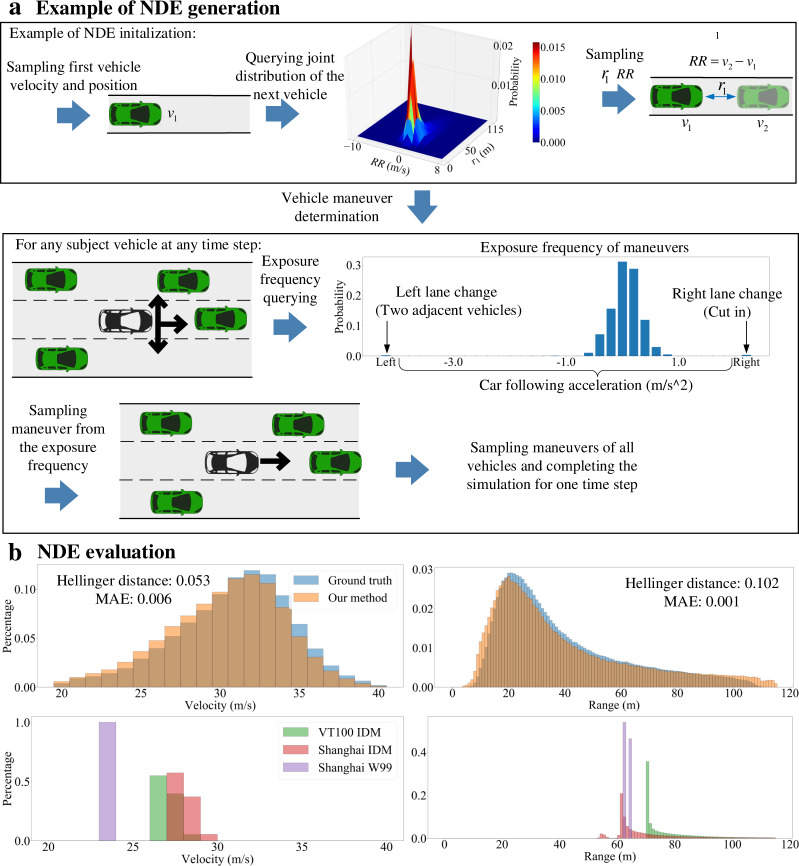
Generation and evaluation of the NDE. **a** Example of the naturalistic driving environment (NDE) generation including initialization (top) and vehicle maneuver determination (bottom). The image of vehicles is previously published at https://commons.wikimedia.org/wiki/File:C3top.png under the Creative Common CC0 1.0 Universal Public Domain Dedication **b** Evaluation results of the generated NDE. Top: the ground truth comes from the distributions of naturalistic driving data, and the distributions of our method come from simulations of the generated NDE. Bottom: simulation results of the intelligent driving model (IDM) model calibrated by the dataset from Virginia and Shanghai, respectively, and the Wiedemann99 model calibrated by the dataset from Shanghai.

The NDE is generated by sampling initial conditions and vehicle maneuvers from the obtained distributions. The goal of initialization is to resemble naturalistic speeds and distances of vehicles as a starting point of the NDE simulation. Toward this goal, the first vehicle of each lane is determined by sampling its position inside an initial zone and its speed from the empirical speed distribution. Then the joint distributions of bumper-to-bumper distances and relative speeds are queried from the obtained empirical distributions so that initial positions and speeds of downstream vehicles can be determined sequentially for each lane (Fig. 3a, top). At each time step of the NDE simulation, vehicle maneuvers are determined by sampling from the empirical distributions of each corresponding maneuver category. For example, as shown in Fig. 3a (bottom), the SV has 33 possible maneuvers: left lane change (with two adjacent vehicles), 31 car following accelerations, and right lane change (in this case it is a cut in). To simplify the sampling process, all vehicles are assumed to select maneuvers independently and simultaneously for each time step. This completes the simulation for one time step (1 s) with all vehicle states updated. The underlying highway traffic simulator^23^ determines specific positions, speeds, and steering angles of all vehicles with bicycle models at a frequency of 15 Hz during each time step. All lane-changing maneuvers are set completed within one time step. The simulation continues until all simulation time steps are completed. An additional explanation of the NDE generation is provided in Supplementary Movie 1.

To evaluate the generated NDE, we compared the distributions of speeds and bumper-to-bumper spaces (range) between the constructed NDE and the ground truth from NDD. We collected data by simulating NDE for about 20,000 kilometers. Figure 3b (top) shows that the generated NDE produces the probabilistic distributions that are very similar to the naturalistic ones. To quantify the similarity, we calculated the Hellinger distance and mean absolute error (MAE). As for comparisons, we also simulated two well-known driving behavior models in the transportation domain, Intelligent Driver Model (IDM)^30^ and Wiedemann99 model, whose parameters were calibrated by the NDD from Virginia^31^ (denoted as VT100 IDM) and Shanghai^32^ (denoted as Shanghai IDM and Shanghai W99), respectively. We collected data by simulating these three models for about 20,000 kilometers, respectively. For fair comparisons, we set the same traffic volumes (about 1360 vehicles per hour per lane) for all simulations and collected data after the warm-up time. As shown in Fig. 3b (bottom), all distributions of these models are significantly abnormal and unnatural, because of the lack of model randomness and flexibility, though we cannot access the specific NDD (the ground truth) from Virginia and Shanghai for quantitative comparisons. This also provides evidence that existing driving behavior models cannot be used directly for the construction of NDE.

### Generation and evaluation of NADE

The most significant part of our method is the generation of NADE for driving intelligence testing of AVs. In essence, we aim to construct new distributions, as the replacement of the naturalistic distributions in NDE, for sampling maneuvers of background vehicles (BVs). The goal is to adjust the maneuvers of BVs intelligently to test the driving intelligence of an AV unbiasedly and efficiently. As our method is based on the importance sampling theory, the new distributions are also denoted as importance functions. To solve the challenge of high dimensionality, we only twist the behavior distributions of the principal other vehicle (POV) at critical moments, while others keep following their naturalistic distributions as in NDE. Because most accidents involve only a small number of vehicles, it is reasonable to identify at most one POV at each moment, and the generalization of our method to multiple POVs is straightforward. In the section of “Methods”, we provide theoretical proof on the unbiasedness and efficiency of the proposed method.

To identify the POV and construct the importance function, at each time step, each BV’s maneuver is evaluated by a newly defined quantity, criticality, which can be computed as a multiplication of exposure frequency and maneuver challenge. The exposure frequency represents the naturalistic probability of the maneuver in NDE, while the maneuver challenge measures its safety challenge to the AV under test. A BV is identified as the POV if its criticality value is largest among all BVs and larger than a threshold. The moment with at least one POV is identified as a critical moment. For the POV at the critical moment, the defensive importance sampling^33^ is adopted, and the importance function is constructed by the weighted average of the exposure frequency and the normalized criticality. By sampling maneuvers of the POVs from the importance functions at critical moments, while keeping other vehicles follow naturalistic distribution at all non-critical times, the resulting NDE becomes both naturalistic and adversarial, i.e., the NADE.

As discussed above, one important step of our method is to calculate the maneuver challenge of each BV’s maneuver at every state. The maneuver challenge is defined as the occurrence probability of a crash accident with the AV under test if the BV takes the maneuver at the state. As the calculation of maneuver challenge involves the interdependency of maneuvers from both the AV and BVs in the following time steps, reinforcement learning or deep reinforcement learning methods with delayed rewards may be used, similar to the use of value networks in AlphaGo^22^. In this paper, we adopted reinforcement learning techniques for basic scenarios such as car-following, while more general scenarios can be approximated by the combination of basic scenarios.

As the specifics of the behavior model of the AV under test are usually unknown, we utilize surrogate models (SMs) to approximate the maneuver challenge. Although approximation errors usually exist, the maneuver challenge can provide valuable information on the impact of BV’s maneuvers. SMs can be constructed based on common knowledge of AVs or prior tests of AVs. In this study, we utilize the IDM and MOBIL (Minimizing Overall Braking Induced by Lane change) models as SMs, which are commonly used in the transportation domain^34^. To capture the uncertainty of AVs, we modify the MOBIL model as a stochastic lane-changing model described in more detail in the Supplementary Methods.

**Fig. 4:**
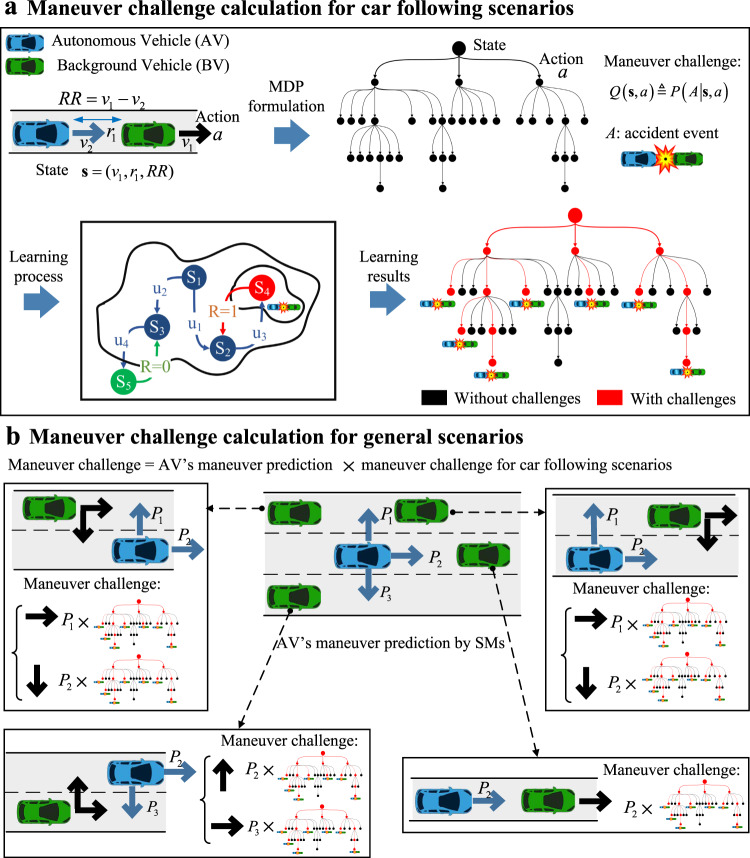
Illustration of maneuver challenge calculation. **a** Maneuver challenge calculation of the BV’s accelerations for car following scenarios based on reinforcement learning techniques. The car following scenarios are formulated based on the Markov decision process (MDP), and the maneuver challenge values are calculated by the learning process. **b** Example of maneuver challenge calculation for general scenarios based on autonomous vehicle (AV)’s maneuver prediction by surrogate models (SMs) and results of car-following scenarios. The image of vehicles is previously published at https://commons.wikimedia.org/wiki/File:C3top.png under the Creative Common CC0 1.0 Universal Public Domain Dedication.

With the SMs, we propose to learn the maneuver challenge for car-following scenarios by the reinforcement learning (RL) method (Fig. 4a, top). Specifically, the state is defined as the BV’s speed, bumper-to-bumper distance, and speed difference, and the action is defined as the BV’s acceleration. Based on MDP, car-following scenarios can be represented by a decision tree, where each branch from the initial state to the terminal state specifies a car-following trajectory. To handle the delayed reward of AV’s accidents, the state-action value of RL is defined as the maneuver challenge, while the reward is set to one for the AV’s accident event and zero for safe states. The states and actions, which may eventually lead to accidents of the AVs, have positive challenge values. Readers can find more technical details^15^. The learning process took only about 20 min to the convergence in a desktop computer equipped with Intel i7-7700 CPU and 16 G RAM.

For general scenarios, we propose to calculate the maneuver challenge for each BV based on the maneuver prediction of the AV and the results of car-following scenarios (Fig. 4b). The basic idea is to calculate the maneuver challenge of each BV at the current time by taking the expectation of its maneuver challenge over all of its possible maneuvers at the next time step. The AV’s maneuvers are predicted as a probability distribution by the SMs. To demonstrate the computation of maneuver challenge, let us take the BV in the top left of Fig. 4b as an example. For the BV, there are two possible maneuvers, one is longitudinal acceleration, and the other is to take the right lane change. For the AV, there are three possible maneuvers: left lane change, longitudinal accelerations, and right lane change. Each of the maneuvers is predicted by the SM with a probability. Between the AV and the BV, there are a total of six possible maneuver combinations, among which two of them are predicted to have non-zero maneuver challenges in the next time step. One is the BV makes right lane change while the AV remains longitudinal, the other is the BV remains longitudinal while the AV makes left lane change. In both scenarios, the BV and the AV are in a car-following situation after the lane-change maneuver, where the maneuver challenge can be obtained with the RL model discussed above. The overall maneuver challenge of the BV is an expectation of those in the two car-following situations.

**Fig. 5:**
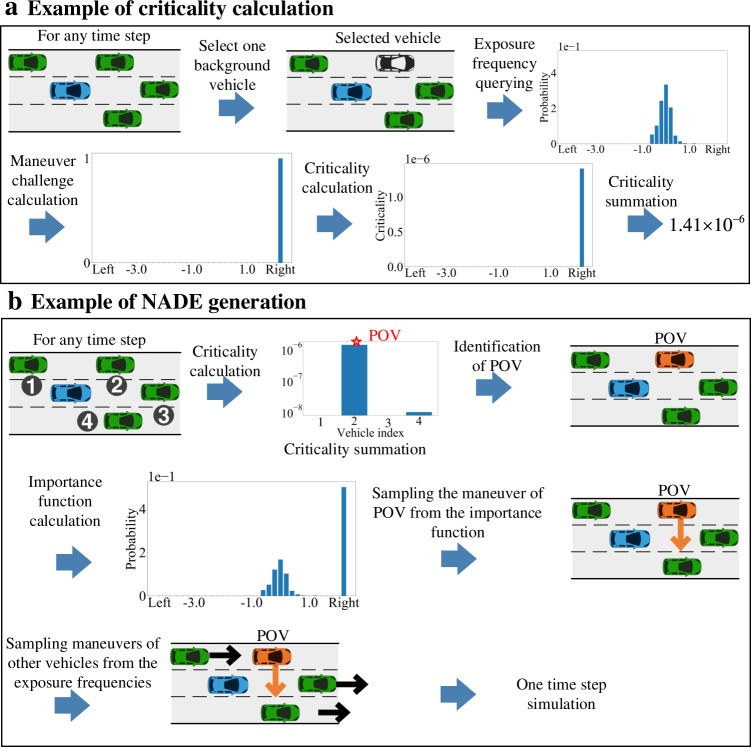
Illustration of NADE generation. **a** Example of criticality calculation. For each background vehicle (green vehicle) at each time step, the criticality of each maneuver is calculated by the multiplication of exposure frequency and maneuver challenge. The blue vehicle denotes the autonomous vehicle. **b** Example of the naturalistic and adversarial driving environment (NADE) generation. For each time step, the criticality summations of all background vehicles are calculated for the identification of the principal other vehicle (POV, orange vehicle). If the POV exists, the importance function is calculated, and the maneuver of the POV is sampled from the importance function, while others follow their naturalistic distributions. The image of vehicles is previously published under the Creative Common CC0 1.0 Universal Public Domain Dedication at https://commons.wikimedia.org/wiki/File:C3top.png.

After calculating the maneuver challenge, the criticality of each BV’s maneuver at each state can be calculated. For example, as shown in Fig. 5a, the exposure frequency of each BV can be queried as in NDE, and the maneuver challenge is calculated as discussed above. Then the criticality is obtained by multiplying the exposure frequency and maneuver challenge. The criticality of most BVs’ maneuvers is zero because either the exposure frequency is zero (impossible maneuver) or the maneuver challenge is zero (unchallenging maneuver).

Among all the BVs surrounding the AV, a BV is identified as the POV if its criticality value is the largest and larger than a threshold (e.g., 0). The moment with a POV is identified as the critical moment. For the POV at the critical moment, the importance functions are constructed by the weighted average of the exposure frequency and the normalized criticality: with the probability *ε*, we sample maneuvers from the exposure frequency, while with the probability 1−*ε*, we sample maneuvers from the normalized criticality. Inspired by the defensive importance sampling, the weighted average can mitigate the influences of the approximation errors of maneuver challenge. The maneuver of POV at the critical moment is then sampled from the importance function, while maneuvers for all other vehicles are sampled from the naturalistic distribution as in NDE. This completes the simulation for one time step (1 s is used in our examples) with all vehicle states updated. The simulation continues until accidents happen or all simulation time steps are completed. Figure 5b shows an example of the NADE generation procedure. An additional explanation of the NADE generation is provided in Supplementary Movie 2.

**Fig. 6:**
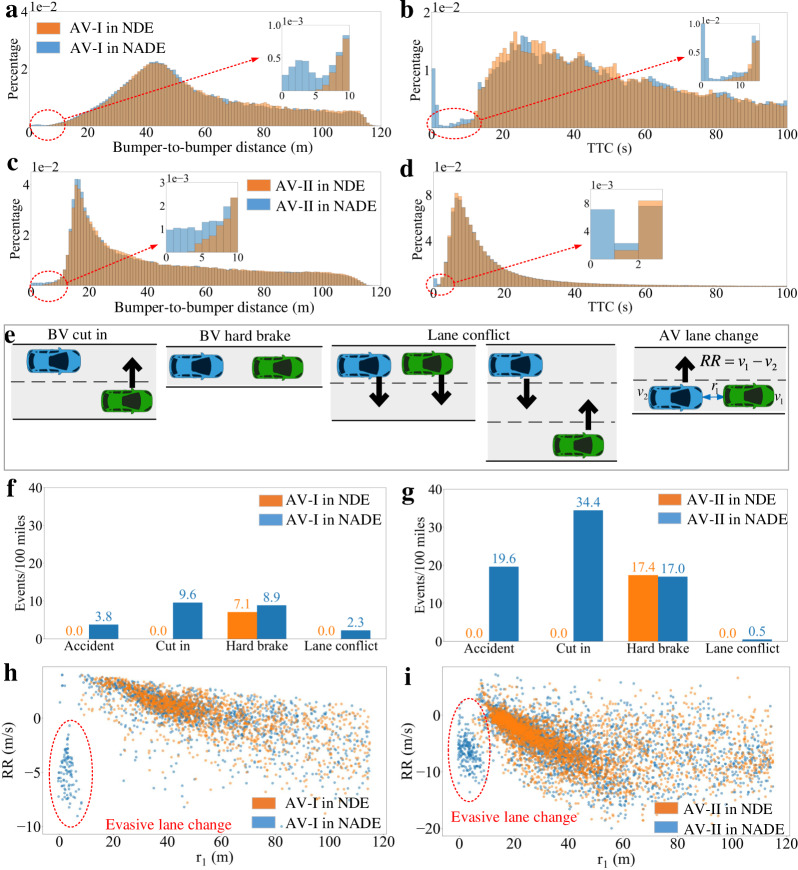
Evaluation of the generated NADE. Distributions of bumper-to-bumper distance (**a**) and TTC (**b**) for the AV-I model. Distributions of bumper-to-bumper distance (**c**) and TTC (**d**) for the AV-II model. **e** Illustration of the events of background vehicle (BV, green vehicle) cut in, BV hard brake, lane conflict, and autonomous vehicle (AV, blue vehicle) lane change. The image of vehicles is previously published under the Creative Common CC0 1.0 Universal Public Domain Dedication at https://commons.wikimedia.org/wiki/File:C3top.png. The number of events encountered by the AV-I model (**f**) and the AV-II model (**g**) for every 100 miles. Distributions of lane changing events of the AV-I model (**h**) and the AV-II model (**i**), where the evasive lane change events are circled by the red dashed lines.

To evaluate the generated NADE, we completed 2000 km simulations of AVs in NDE and NADE, respectively, and calculated the distributions of bumper-to-bumper spaces and time-to-collision (TTC) for AVs. To investigate the influences of AVs, we developed two different types of AV models: the AV-I model was constructed based on IDM and MOBIL, while the AV-II model was trained by deep reinforcement learning (DRL) techniques considering both efficiency and safety. More details on AV-I and AV-II can be found in the Supplementary Methods. Figure 6a, b shows that, for the AV-I model, NADE generates very similar distributions as NDE (naturalistic), but much more dangerous scenarios with small distances and TTC (adversarial). It is also true for the AV-II model, as shown in Fig. 6c, d. The results also indicate that the AV-II model is more aggressive than the AV-I model, because the AV-II model has smaller bumper-to-bumper distances and TTC in NDE. This is not surprising because IDM and MOBIL are designed to be collision-free so AV-I is comparatively conservative.

We also compared the events encountered by the AVs in NDE and NADE. Besides the accident event, we defined the events of BV cut-in, BV hard brake, lane conflict, and AV lane change, as shown in Fig. 6e. We queried these events with the following criteria, respectively: (a) a BV cuts in the AV within 1.5 s time headway (THW); (b) a leading BV within 1.5 s THW brakes harder than −3 ms^−2^; (c) the AV and BV are within 1.5 s THW and change to the same lane simultaneously; (d) the AV changes its lane to avoid the front BV. As shown in Fig. 6(f, g), comparing with NDE, NADE generates many more events of the accident, BV cut-in, and lane conflict, and a similar number of BV hard brake events, for both the two AV models. Actually, NDE has no event of accident, BV cut in, and lane conflict in the 2000 km simulations for both the AVs, because of the rareness of these events. Moreover, as shown in Fig. 6h, i, NADE generates much more evasive lane change maneuvers of both the AVs with small relative distances (*r*_1_) and speed differences (*RR*). All these results show that NADE can test the AVs much more effectively by more valuable events, comparing with NDE.

We further investigated the adjustment frequency of BVs’ maneuvers in NADE. Results show that, for every driving mile of the two AVs, we adjusted average of 6.51 and 5.43 times, respectively. As a comparison, there are a total of 381.27 and 351.01 BVs’ maneuvers in the neighborhood (the closest eight vehicles within 120 m) of the AVs every mile. Therefore, we only adjust about 1.7% and 1.5% maneuvers of the environment, which is very sparse and thus keep the environment naturalistic. It validates that sparse but intelligent adjustment of NDE can significantly improve test effectiveness.

### Accuracy and efficiency of driving intelligence testing in NADE

The accuracy and efficiency of driving intelligence test in NADE are theoretically guaranteed and validated in our simulation. To measure the driving intelligence regarding safety, accident rates of the AVs in NDE are utilized as the benchmark. As the NDE is generated based on NDD, it can represent the safety performance of the AVs in the real world. In our experiments, we compared the estimated accident rates and required numbers of tests for both NDE and NADE. For the convenience of experiments, we conducted one simulation test for a constant driving distance (400 m) of the AVs, recorded the test results (accident or not) of the AVs, and calculated the accident rate per simulation test. As the distance of each test is constant, it can easily transform between the accident rate per simulation test and the driving distance. More details can be found in the “Methods” section. To investigate the influences of AV models, both the AV-I and the AV-II models were tested.

**Fig. 7:**
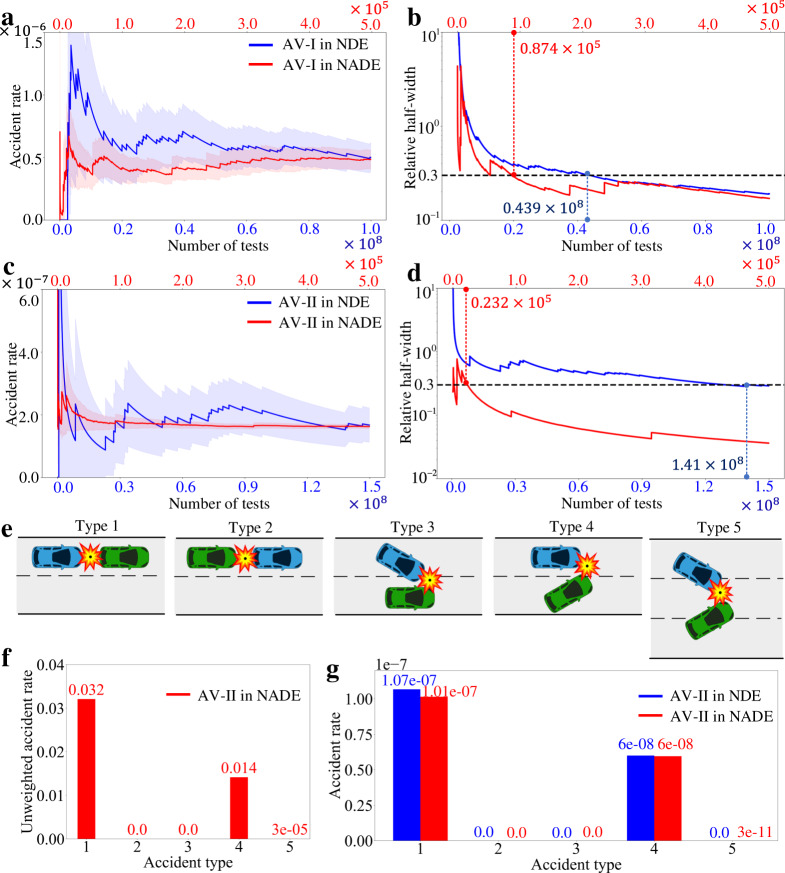
Evaluation accuracy and efficiency for the two AVs by NDE and NADE. The accident rate estimation of the AV-I model (**a**) and the AV-II model (**c**). The relative half-width of the AV-I model (**b**) and the AV-II model (**d**). **e** Illustration of the five accident types. Type 1: the autonomous vehicle (AV, blue vehicle) has a rear-end collision with the background vehicle (BV, green vehicle). Type 2: the BV has a rear-end collision with the AV. Type 3: the AV makes a lane change and has a sideswipe collision with the BV. Type 4: the BV makes a lane change and has a sideswipe collision with the AV. Type 5: both the AV and BV make a lane change and have a sideswipe collision. The image of vehicles is previously published at https://commons.wikimedia.org/wiki/File:C3top.png under the Creative Common CC0 1.0 Universal Public Domain Dedication. **f** Unweighted accident rate of each type for the AV-II model in the naturalistic and adversarial driving environment (NADE). **g** Accident rate of each type in the naturalistic driving environment (NDE) and weighted accident rate of each type in NADE.

Figure7a–d shows the evaluation results of the accident rate per test for both the AVs in NDE and NADE. The blue line represents the results of testing in NDE, and the bottom *x*-axis indicates the number of tests. The red line represents the results in NADE, and the top *x*-axis for the number of tests. The light shadow represents the 90% confidence level. As shown in Fig. 7a, c, NADE obtains the same accident rate estimation with NDE by a much smaller number of tests for both the AVs. We further calculated the average driving distance per accident, which were 5.13 × 10^5^ and 1.54 × 10^6^ miles per accident. As human drivers in the US have on average 4.79 × 10^5^ miles between two accidents on highway^35^, the AV-I model has similar safety performance with human drivers, while the AV-II model is better.

To measure the efficiency, we calculated the relative half-width (RHW)^12^ as the measurement of evaluation precision and calculated the minimum number of tests for reaching a pre-determined precision threshold (RHW is 0.3). As shown in Fig. 7b, for the AV-I model, NADE requires only 8.74 × 10^4^ number of tests, while NDE requires 4.39 × 10^7^ number of tests. Our method can accelerate the evaluation for about 500 times and reduce about 10 million driving miles. Similarly, for the AV-II model, NADE requires the 2.32 × 10^4^ number of tests, while NDE requires 1.41 × 10^8^ number of tests, as shown in Fig. 7d. Our method can accelerate the evaluation for about 6,000 times and eliminate 35 million driving miles.

To investigate the influences of parameters in NADE, we further conducted the sensitivity analysis of *ε*, which was used in constructing important functions. For each value (0.1, 0.3, and 0.5), we completed the tests in NADE and calculated the minimum number of tests for reaching the precision threshold. To mitigate the randomness of the results, we repeated the tests 10 times, and calculated the average minimum number of tests, as shown in Table 1. Please note that NDE is equivalent to NADE with *ε* = 1. Results show that NADE can improve the evaluation efficiency significantly for all three values. The best result is obtained for the AV-I model with *ε* = 0.5 and AV-II model with *ε* = 0.3. As discussed before, the introduction of *ε* is to mitigate the influence of approximation errors of maneuver challenges. As the approximation errors may be different for different AVs, the optimal value of *ε* and the optimal acceleration rates are different. In practice, *ε* = 0.5 is a good choice balancing the optimality and the robustness.

**Table 1:** The average minimum number and average wall-clock time of tests in the naturalistic driving environment (NDE) and the naturalistic and adversarial driving environment (NADE) with different parameters *ε* = 0.1, 0.3, 0.5 for reaching the precision threshold (RHW is 0.3).

Autonomous vehicle		NADE (*ε* = 0.1)	NADE (*ε* = 0.3)	NADE (*ε* = 0.5)	NDE (*ε* = 1.0)
AV-I	No. of tests	1.85 × 10^5^	1.52 × 10^5^	1.14 × 10^5^	4.39 × 10^7^
AV-I	Time (s)	324.61	268.14	196.82	6.89 × 10^4^
AV-II	No. of Tests	9.40 × 10^3^	2.27 × 10^3^	6.01 × 10^3^	1.41 × 10^8^
AV-II	Time (s)	17.25	4.06	10.66	2.33 × 10^5^

To investigate the computational cost of NADE, we also compared the average wall-clock time used by NDE and NADE for reaching the precision threshold. We conducted the simulations of NDE and NADE on the University of Michigan’s Great Lakes High-Performance Computing (HPC) cluster using 500 cores (Intel Xeon Gold 6154 processor) and 2500 GB RAM. As shown in Table 1, the tests in NADE reduce the computational time significantly for both AV models with all three values of *ε*.

To validate the unbiasedness about accident types, we adopted the crash type diagram defined by the Fatality Analysis Reporting System (FARS)^36^, which is a nationwide census provided by National Highway Traffic Safety Administration (NHTSA) for data regarding fatal injuries suffered in motor vehicle traffic crashes. For the highway driving case in this paper, we only have the accidents between the AV and BVs, so the five accident types are identified, as shown in Fig. 7e. We note that accident type 1 can also be caused by reckless cut-in of the BV, where the difference from type 4 is that the AV collides with the BV from the rear end. We have compared the results for the AV-II model in NDE and NADE. Figure 7f shows the unweighted accident rate of each type in NADE. As NADE is more adversarial than NDE, the total accident rate is 0.046 accidents per simulation test, which is much larger than NDE. As required by the importance sampling theory, each accident event should be weighted by the likelihood ratio (see the “Methods” section) to keep the unbiasedness. Figure 7g shows that the weighted accident rates for all five types are the same as the results in NDE within the evaluation precision (the relative half-width is smaller than 0.3). The summation of the accident rates of all five types is the same as the total accident rate, so these five types include all accidents of the AV-II model in both NDE and NADE.

### Adversarial examples in NADE

**Fig. 8:**
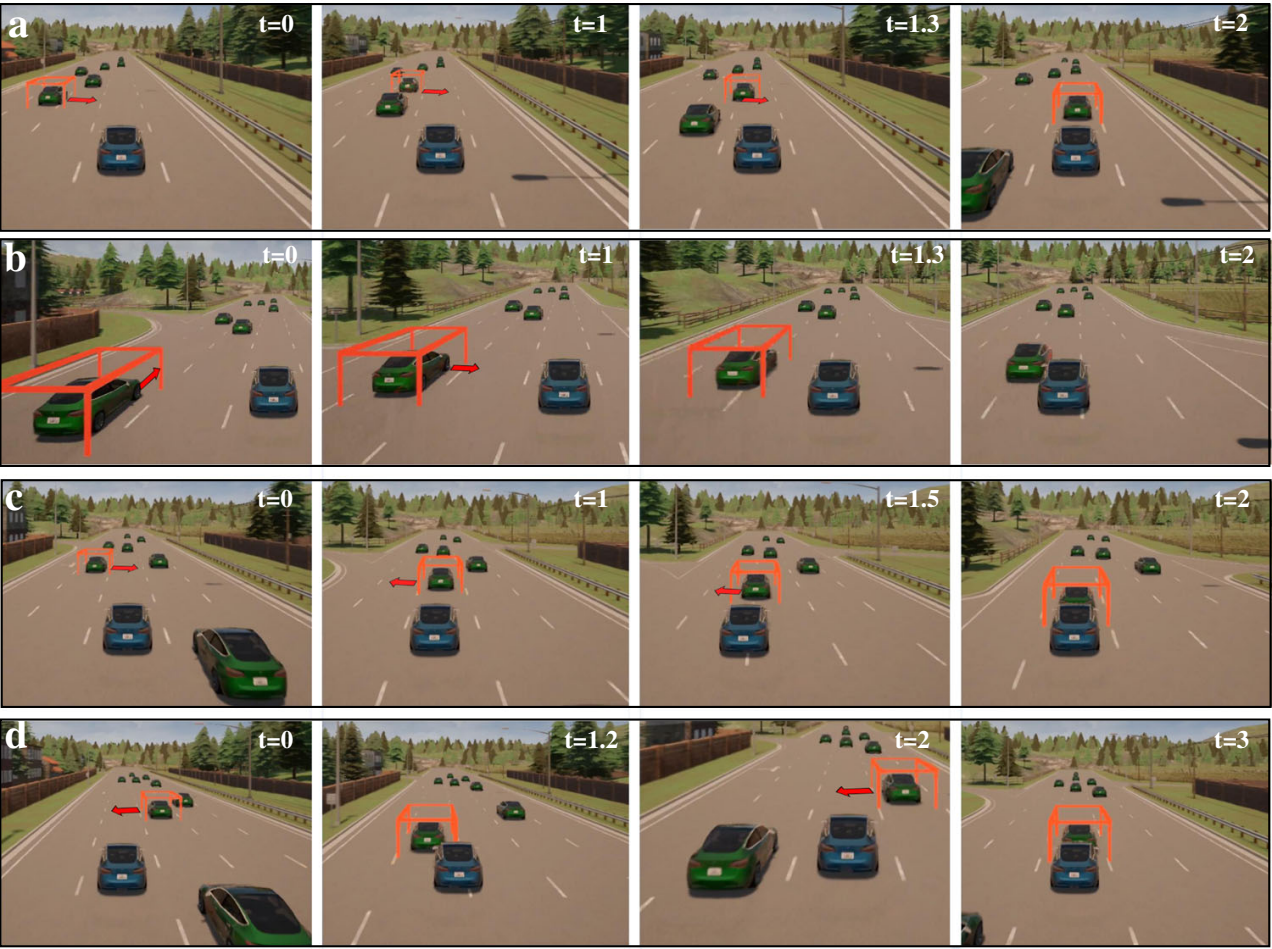
Adversarial examples generated in NADE. **a** The autonomous vehicle (AV, blue vehicle) was on high speed and had a rear-end collision with the cut-in principal other vehicle (POV, green vehicle with orange rectangle) after two front-left POVs sequentially changed their lanes towards the AV. **b** The AV made a left lane change and collided with the POV due to a lane conflict (the POV accelerated first and then made a right lane change, simultaneously with the AV). **c** The AV turned left to avoid the collision to the cut-in POV but failed as the cut-in POV switched back to the left lane simultaneously with the AV. **d** The AV made an evasive lane change to avoid one cut-in POV but eventually collided with another cut-in POV.

We investigated the capability of NADE for generating adversarial examples. Adversarial examples have been widely investigated in the domain of machine learning. By applying small but intentional perturbations to examples from the dataset, adversarial examples can cause severe failures to many machine-learning methods and, therefore, provide insights for further improvement^37^. Similarly, adversarial examples, sometimes known as corner cases, edge cases, or worst cases, play an important role in the development and evaluation of AVs. As they happen rarely in the NDE, it is significant to generate adversarial examples systematically. As demonstrated above, the NADE can generate many more accidents than the NDE. The key is to identify cases that are valuable and informative. We propose two criteria as examples to illustrate the potential of NADE for generating adversarial examples. The first is the simulation weight, which is the likelihood ratio of the simulation test. A smaller simulation weight usually indicates a higher probability of the test to be an adversarial example. The second is the diversity of the events (as defined in Fig. 6e) involved during the test. A test involving diverse events usually contains more information for understanding the AV model under test. Figure 8 provides several examples identified using the above criteria. The blue vehicle represents the AV under test, the green vehicles represent the BVs, and the green vehicle with the orange rectangle represents the POV. An additional explanation of these adversarial examples is provided in Supplementary Movie 3.

## Discussion

In the previous section, we showed the effectiveness of NADE for driving intelligence testing of AVs. Our method can be used to enhance the existing life-like simulations to accelerate the test process. It can also be used to systematically generate valuable adversarial examples for the further development of AVs. The adversarial yet naturalistic environment is also promising for accelerated training of AVs. The scalability of our method makes it possible to be used in large-scale simulations, such as a city-scale driving environment, as long as sufficient naturalistic driving data is available. The NADE framework may also be applied to the intelligence tests of other types of robotics with similar features.

The efficiency of using NADE for driving intelligence testing is dependent on the approximation error of the maneuver challenge of BVs. The approximation error comes from two problems, one is the difference between the SM and the real AV under test, and the other is the prediction error of the AV maneuver in the following time steps, which is interdependent on the maneuvers of BVs. The first problem can be mitigated by prior knowledge of the AV, such as the testing results of its previous model. Although this knowledge may not be complete, it can be leveraged by our framework in constructing SM and thus reduces the difference between the SM and the AV model. The second problem is essentially a policy evaluation in the AI domain, where state-of-the-art algorithms such as those from deep reinforcement learning can be utilized for further reducing the approximation errors. As discussed before, with smaller approximation errors, the NADE can further accelerate the testing process of AVs.

Our approach requires a large amount of naturalistic driving data to model the driving behaviors of background vehicles in NDE. The relative position and speed information of the ego vehicle and surrounding vehicles are needed to construct the empirical distributions of vehicle interaction behaviors. For a complex driving environment, millions of data points would be required to represent the variability of the environment. Fortunately, with the deployment of vehicle-based and infrastructure-based perception sensors, nowadays the data can be collected at a lower cost and become more accessible^38^.

The case study in this paper has several simplifications (e.g., highway driving, limited actions, vehicles only, etc.) for the convenience of experiments. However, as shown in the “Methods” section, our approach is not limited to these simplifications and can be readily extended for more complex scenarios, larger action space, and various road users, with the input of sufficient naturalistic driving data.

Another limitation of our approach is the lack of perception related tests (e.g., weather conditions) in the generated NADE. However, if the challenge to AVs’ perception can be measured and a small but critical set of variables regarding AVs’ perception can be identified and adjusted, NADE for perception related tests can also be constructed. There have been significant advances in adversarial image synthesis^39–41^, which are promising for solving this problem.

## Methods

### Generation of NDE

This section describes our data-driven algorithm for NDE construction, which, in essence, is a sampling process from the joint distributions of the variables that represent the complexity and variability of NDE. To simplify the high dimensional spatiotemporal distributions, the NDE is modeled with the Markov decision process (MDP) and probabilistic graphical models (PGM), leveraging spatiotemporal independence relations among the variables. Specifically, the NDE is decomposed into six different scenarios (Fig. 2c), and, for each scenario, the exposure frequency distribution of each vehicle maneuver is calculated from the NDD. The NDE can then be simulated by sampling each vehicle maneuver from the obtained exposure frequency distributions.

In this paper, the NDE is represented by a list of parameters that are pre-determined by the operational design domain (e.g., road type, weather condition, etc.) and variables that may vary (e.g., accelerations of background vehicles). The variables can be represented as

1 $${\mathbf{x}} = \left[ {\begin{array}{*{20}{c}} {{\mathbf{x}}_{1,1}} & \cdots & {{\mathbf{x}}_{1,T}} \\ \vdots & \ddots & \vdots \\ {{\mathbf{x}}_{N,1}} & \cdots & {{\mathbf{x}}_{N,T}} \end{array}} \right],{\mathbf{x}} \in {\mathbf{X}},$$

where **x**_*i,j*_ denoted the variables (e.g., position and speed) of the *i*-th BV at the *j*-th time step, *N* denotes the number of BVs of interest, *T* denotes the total number of time steps, and **X** denotes the feasible space of variables. The NDE generation is to sample values of variables according to their naturalistic joint distributions, denoted as **x** ∼ *P*(**x**).

As *P*(**x**) is extremely high dimensional, we simplify the problem by exploiting spatiotemporal independence relations among the variables. Assuming the Markovian property, the joint distribution can be simplified in a factorized way as

2 $$P({\mathbf{x}}) = P\left( {{\mathbf{s}}\left( 0 \right)} \right) \times \mathop {\prod}\limits_{k = 0}^T {P\left( {{\mathbf{u}}\left( k \right)|{\mathbf{s}}\left( k \right)} \right)} .$$

Here, the state and action at the time step $k = 0, \cdots ,T$ are denoted as

3 $$\begin{array}{l}{\mathbf{s}}\left( k \right) = \left[ {{\mathbf{s}}_0\left( k \right),{\mathbf{s}}_1\left( k \right) \cdots ,{\mathbf{s}}_N\left( k \right)} \right],\\ \quad {\mathbf{u}}(k) = \left[ {{\mathbf{u}}_1\left( k \right), \cdots ,{\mathbf{u}}_N\left( k \right)} \right],\end{array}$$

where **s**_0_ denotes the state (e.g., position and speed) of the AV under test, **s**_*i*_ (*i* = 1,…,*N*) denotes the state of the *i*-th BV, and **u**_*i*_ denotes the maneuver (e.g., longitudinal accelerations) of the *i*-th BV. Then the NDE is generated by sampling maneuvers as **u** (*k*) ~ *P*(**u** (*k*)|**s** (*k*)) at each time step. To simplify *P*(**u** (*k*)|**s** (*k*)), it is assumed that all BVs choose their maneuvers simultaneously and independently, so we can calculate it in a factorized way as

4 $$P\left( {{\mathbf{u}}\left( k \right)|{\mathbf{s}}\left( k \right)} \right) = \mathop {\prod}\limits_{i = 1}^N {P\left( {{\mathbf{u}}_i\left( k \right)|{\mathbf{s}}\left( k \right)} \right)} .$$

The *P*(**u**_*i*_ (*k*)|**s** (*k*)) is further simplified by assuming spatial independence, for example, the car-following maneuvers of a BV are only dependent on states of itself and its leading vehicle. Let *N*_*i*_ denote all vehicles that have dependencies with the *i*-th BV. Then the *P*(**u**_*i*_ (*k*)|**s** (*k*)) can be approximated by $P\left( {{\mathbf{u}}_i\left( k \right)|{\mathbf{s}}_{N_i}\left( k \right)} \right)$.

Finally, the $P\left( {{\mathbf{u}}_i\left( k \right)|{\mathbf{s}}_{N_i}\left( k \right)} \right)$ is calculated by the empirical probability of the state-action pair in NDD, as shown in Fig. 2d.

### Generation of NADE

This section describes our algorithm for NADE construction. The key is to obtain new behavioral distributions *q*(**u**|**s**) as the replacement of *P*(**u**|**s**) in NDE. To overcome the challenge of high dimensionality, we identify the POV at the critical moment and only adjust its behaviors.

To identify POV, we define the maneuver criticality as the multiplication of exposure frequency *P*(**u**_*i*_|**s**) and maneuver challenge *P*(*A*_*i*_|**s**,**u**_*i*_) as

5 $$V\left( {{\mathbf{u}}_i\left| {\mathbf{s}} \right.} \right) \buildrel \Delta \over = P\left( {{\mathbf{u}}_i\left| {\mathbf{s}} \right.} \right) \times P\left( {A_i\left| {{\mathbf{s}},{\mathbf{u}}_i} \right.} \right),$$

where *A*_*i*_ denotes the accident between the *i*-th BV and the AV under test. The first part on the right-hand side is the exposure frequency obtained from NDD. The second part is the maneuver challenge that indicates the accident probability given the state-action pair (**s**, **u**_*i*_). Since we treat the AV model under test as a black box, to approximate the maneuver challenge, we construct SMs of AVs by meta-models, described in more detail in the Supplementary Methods. We should note that the SMs can also be constructed based on the preliminary AV models, so it provides an elegant way to leverage the existing testing results of preliminary AV models. Let *S*_*i*_ denote the accident between the *i*-th BV and the SMs. Then, the maneuver challenge can be approximated by

6 $$\begin{array}{l}P\left( {A_i\left| {{\mathbf{s}},{\mathbf{u}}_i} \right.} \right) = \mathop {\sum}\limits_{{\mathbf{u}}_0} {P\left( {{\mathbf{u}}_0\left| {\mathbf{s}} \right.} \right)P\left( {A_i\left| {{\mathbf{s}},} \right.{\mathbf{u}}_i,{\mathbf{u}}_0} \right)} ,\\ \quad \quad \quad \quad \;\; \approx \mathop {\sum}\limits_{{\mathbf{u}}_0} {P\left( {{\mathbf{u}}_0\left| {\mathbf{s}} \right.} \right)P\left( {S_i\left| {{\mathbf{s}}_{\bar N_i},} \right.{\mathbf{u}}_i,{\mathbf{u}}_0} \right)} ,\end{array}$$

where *P*(**u**_0_|**s**) denotes the probability of the AV’s maneuver **u**_0_ at the state **s**, and ${\mathbf{s}}_{\bar N_i}$ denotes the states of the vehicles that influence the event *S*_*i*_. The first term *P*(**u**_0_|**s**) can be predicted approximately by the SMs, and the second term $P\left( {S_i\left| {{\mathbf{s}}_{\bar N_i},} \right.{\mathbf{u}}_i,{\mathbf{u}}_0} \right)$ can be evaluated by simulations of the SMs in the scenarios specified by $\left( {{\mathbf{s}}_{\bar N_i},{\mathbf{u}}_i,{\mathbf{u}}_0} \right)$. Realizing that the evaluation of $P\left( {S_i\left| {{\mathbf{s}}_{\bar N_i},} \right.{\mathbf{u}}_i,{\mathbf{u}}_0} \right)$ may not be completed by one-time-step simulation, to obtain the evaluation result quickly, reinforcement learning or deep reinforcement learning methods may be used. In this paper, we adopted reinforcement learning techniques for the basic scenarios such as car-following, while more general scenarios can be approximated by the combination of basic scenarios, as shown in Fig. 4.

The criticality for each BV can then be calculated as the summation of maneuver criticality over all the BV’s maneuvers:

7 $$C_i({\mathbf{s}}) \buildrel \Delta \over = \mathop {\sum}\limits_{{\mathbf{u}}_i} {V({\mathbf{u}}_i|{\mathbf{s}})} ,$$

and the POV can be identified by

8 $$c \buildrel \Delta \over = {\mathrm{arg}}\,{\mathrm{max}}_iC_i\left( {\mathbf{s}} \right),$$

if C_*c*_ (*s*) > *C*, where *C* is a pre-determined threshold (e.g., 0). We define the moment as the critical moment if there is at least one POV. Because most accidents involve only two vehicles, we considered at most one POV at each moment in this work. The generalization of this work to multiple POVs is straightforward.

Finally, we construct the importance function *q*(**u**|**s**) by adjusting the maneuvers of POV at the critical moment as

9 $$q\left( {{\mathbf{u}}|{\mathbf{s}}} \right) = q\left( {{\mathbf{u}}_c|{\mathbf{s}}} \right) \times \mathop {\prod}\limits_{i = 1,i \ne c}^N {P\left( {{\mathbf{u}}_i|{\mathbf{s}}} \right)} ,$$

where **u**_*c*_ denotes the maneuver of POV. Only the POV’s maneuver is adjusted by *q*(**u**_*c*_|**s**), while other vehicles follow their naturalistic distributions as in NDE. For uncritical moments, all vehicles behave as in NDE. The *q*(**u**_*c*_|**s**) is constructed by the weighted average of the naturalistic distribution and the normalized criticality distribution as

10 $$q\left( {{\mathbf{u}}_c|{\mathbf{s}}} \right) = \varepsilon P\left( {{\mathbf{u}}_c|{\mathbf{s}}} \right) + \left( {1 - \varepsilon } \right)\frac{{V\left( {{\mathbf{u}}_c|{\mathbf{s}}} \right)}}{{C_c\left( {\mathbf{s}} \right)}},$$

where *ε* > 0 is the weight of the naturalistic distribution. It can balance the exploitation and exploration to mitigate the influence of approximation errors of maneuver criticality.

### Evaluation of AVs with NADE

This section describes how to estimate the accident rate of AVs when testing with NADE. Specifically, if the event of interest (accident event of AVs in this paper) is denoted as *A*, we can measure the driving intelligence of AVs by

11 $$P\left( A \right) = \mathop {\sum}\limits_{{\mathbf{x}} \in {\mathbf{X}}} {P\left( {A|{\mathbf{x}}} \right)P\left( {\mathbf{x}} \right),}$$

where **x** denotes variables of the driving environment, and **X** denotes its feasible domain. The NDE-based testing method is essentially to estimate *P*(*A*) by the Crude Monte Carlo (CMC) method as

12 $$\begin{array}{c}P\left( A \right) \approx \frac{1}{n}\mathop {\sum}\limits_{i = 1}^n {P\left( {A|{\mathbf{x}}_i} \right)} ,{\mathbf{x}}_i \sim P\left( {\mathbf{x}} \right),\\ \approx \frac{m}{n},\end{array}$$

where *n* denotes the number of tests, *m* the number of the event *A* during the tests, and **x**_*i*_ ∼ *P*(**x**) indicates that the variables are sampled from their naturalistic distributions.

Because the event *A* is usually a rare event for AVs in NDE, the CMC method suffers from severe inefficiency limitations. To mitigate this issue, the importance sampling (IS) method was applied for scenario-based methods as

13 $$\begin{array}{c}P\left( A \right) = \mathop {\sum}\limits_{{\mathbf{x}} \in {\mathbf{X}}} {\frac{{P\left( {A|{\mathbf{x}}} \right)P\left( {\mathbf{x}} \right)}}{{q\left( {\mathbf{x}} \right)}}q\left( {\mathbf{x}} \right),} \\ \approx \frac{1}{n}\mathop {\sum}\limits_{i = 1}^n {\frac{{P\left( {A|{\mathbf{x}}_i} \right)P\left( {{\mathbf{x}}_i} \right)}}{{q\left( {{\mathbf{x}}_i} \right)}}} ,{\mathbf{x}}_i \sim q\left( {\mathbf{x}} \right),\end{array}$$

where *q*(**x**) is called the importance function. By introducing importance functions, the testing priority of critical scenarios will be improved, so does the evaluation efficiency^14–17^. However, all existing IS-based methods suffer from the “curse of dimensionality”^19^, and thus cannot be applied directly for the complex driving environment.

We solve the “curse of dimensionality” by combining CMC and IS methods. Conceptually, only the critical variables are adjusted by the IS method, while other variables keep their naturalistic distributions following the CMC method. Following the formulation and assumptions in NDE and NADE, we derive the performance estimation equation as

14 $$P\left( A \right) \approx \frac{1}{n}\mathop {\sum}\limits_{i = 1}^n {\left( {P\left( {A|{\mathbf{x}}_i} \right) \times \left[ {\mathop {\prod}\limits_{k = 1}^{T_i} {\frac{{P\left( {{\mathbf{u}}\left( k \right)|{\mathbf{s}}\left( k \right)} \right)}}{{q\left( {{\mathbf{u}}\left( k \right)|{\mathbf{s}}\left( k \right)} \right)}}} } \right]} \right)} ,$$

where *T*_*i*_ denotes the total time steps of the *i*-th simulation test. In this study, we terminate a test if an event *A* happens or the test reaches the pre-determined driving distance. Denote *T*_*i,c*_ as the set of critical moments of the *i*-th test, and, finally, the performance estimation equation can be obtained as

15 $$P\left( A \right) \approx \frac{1}{n}\mathop {\sum}\limits_{i = 1}^n {\left( {P\left( {A|{\mathbf{x}}_i} \right) \times \left[ {\mathop {\prod}\limits_{k \in T_{i,c}} {R\left( k \right)} } \right]} \right)} ,$$

where

16 $$R\left( k \right) \buildrel \Delta \over = \frac{{P\left( {{\mathbf{u}}_c\left( k \right)|{\mathbf{s}}\left( k \right)} \right)}}{{q\left( {{\mathbf{u}}_c\left( k \right)|{\mathbf{s}}\left( k \right)} \right)}},$$

is the simulation weight (likelihood ratio) recorded during the test process. The *P*(*A*|**x**_*i*_) is estimated by counting the number of accident events occurring in the test. Based on this equation, the accident rate of the AV under test can be estimated by the testing results in NADE.

### Theoretical analysis of accuracy and efficiency

This section theoretically justifies the accuracy and efficiency of our NADE-based testing method. As proved by the IS theory^33^, the performance evaluation is unbiased if *q*(**x**) > 0 whenever *P*(*A*|**x**)*P*(**x**) ≠ 0. As *ε* > 0 in the generation of NADE, we can guarantee *q*(**u**|**s**) > 0 whenever *P*(**u**|**s**) ≠ 0 for all states and actions, which is sufficient for unbiasedness. Therefore, our NADE-based testing method is statistically accurate.

To justify the efficiency of our method, we introduce the lemma regarding the “curse of dimensionality” of the IS method^19^:

#### Lemma 1.

The estimation variance of the IS method has the lower bound as

17 $$\sigma ^2 \ge P^2\left( A \right)\left\{ {\exp \left[ {D_{{\mathrm{KL}}}\left( {q^ \ast \left( {\mathbf{x}} \right)\parallel q\left( {\mathbf{x}} \right)} \right)} \right] - 1} \right\},$$

where q^*^(**x**) is the optimal importance function with zero estimation variance, and

18 $$D_{{\mathrm{KL}}}\left( {q^ \ast \left( {\mathbf{x}} \right)\parallel q\left( {\mathbf{x}} \right)} \right) = E_{q^ \ast \left( {\mathbf{x}} \right)}\left( {\log \frac{{q^ \ast \left( {\mathbf{x}} \right)}}{{q\left( {\mathbf{x}} \right)}}} \right),$$

is the Kullback–Leibler (KL) divergence as the measurement of discrepancies between q^*^(**x**) and *q*(**x**).

Following the independence assumptions in NDE, if the IS method is directly applied, we can derive the equations as

19 $$q^ \ast \left( {\mathbf{x}} \right) = q^ \ast \left( {{\mathbf{s}}\left( 0 \right)} \right) \times \mathop {\prod}\limits_{k = 1}^T {q^ \ast \left( {{\mathbf{u}}\left( k \right)|{\mathbf{s}}\left( k \right)} \right)} ,$$

20 $$\log \frac{{q^ \ast \left( {\mathbf{x}} \right)}}{{q\left( {\mathbf{x}} \right)}} = \log \frac{{q^ \ast \left( {{\mathbf{s}}\left( 0 \right)} \right)}}{{q\left( {{\mathbf{s}}\left( 0 \right)} \right)}} + \mathop {\sum}\limits_{k = 1}^T {\mathop {\sum}\limits_{i = 1}^N {\log \frac{{q^ \ast \left( {{\mathbf{u}}_i\left( k \right)|{\mathbf{s}}\left( k \right)} \right)}}{{q\left( {{\mathbf{u}}_i\left( k \right)|{\mathbf{s}}\left( k \right)} \right)}}} } .$$

As $\log \frac{{q \ast ({\mathbf{u}}_i(k)|{\mathbf{s}}(k))}}{{q({\mathbf{u}}_i(k)|{\mathbf{s}}(k))}}$ is usually predetermined by prior knowledge utilized for generating the importance functions, the KL divergence will increase linearly with the dimensionality (O(*NT*)), and, therefore, the estimation variance will increase exponentially with the dimensionality, leading to the “curse of dimensionality”.

For NADE, if the variance is only dependent on the dimensionality of the adjusted critical variables, i.e., the maneuvers of POV at the critical moments, then our method addresses the “curse of dimensionality”. Specifically, if we denote *x*_*c*_ the critical variables, which are independent of all other variables *x*_−*c*_, we propose the theorem as follows, and the proof can be found in the Supplementary Methods.

#### Theorem 1:

The estimation variance of our method has the following relations:

21 $$\begin{array}{c}\sigma ^2 = P^2\left( A \right)D_{\chi ^2}\left( {q^ \ast \left( {{\mathbf{x}}_c} \right)\parallel q\left( {{\mathbf{x}}_c} \right)} \right) + D\left( {{\mathbf{x}}_c\parallel {\mathbf{x}}} \right)\\ \ge P^2\left( A \right)\left\{ {\exp \left[ {D_{KL}\left( {q^ \ast \left( {{\mathbf{x}}_c} \right)\parallel q\left( {{\mathbf{x}}_c} \right)} \right)} \right] - 1} \right\} + D\left( {{\mathbf{x}}_c\parallel {\mathbf{x}}} \right)\end{array}$$

where $D_{\chi ^2}\left( {q^ \ast ({\mathbf{x}}_c)\parallel q({\mathbf{x}}_c)} \right) = E_{q\left( {{\mathbf{x}}_c} \right)}\left( {\left( {\frac{{q^ \ast ({\mathbf{x}}_c)}}{{q({\mathbf{x}}_c)}} - 1} \right)^2} \right)$ denotes the *χ*^2^-divergence, $q^ \ast \left( {{\mathbf{x}}_c} \right) = \frac{{P\left( {A\left| {{\mathbf{x}}_c} \right.} \right)P\left( {{\mathbf{x}}_c} \right)}}{{P\left( A \right)}}$ denotes the optimal importance function for the critical variables, and $D({\mathbf{x}}_c\parallel {\mathbf{x}}) = E_{q({\mathbf{x}})}\left[ {\left( {P(A|{\mathbf{x}}) - P(A|{\mathbf{x}}_c)} \right)^2\frac{{P^2({\mathbf{x}})}}{{q^2({\mathbf{x}})}}} \right]$ measures how critical the adjusted variables are.

The term *D*(**x**_*c*_||**x**) measures the variance caused by the identification of critical variables. The more critical the adjusted variables **x**_*c*_ are, the closer *P*(*A*|**x**_*c*_) is to *P*(*A*|**x**), and thus the closer *D*(**x**_*c*_||**x**) is to zero.

The KL divergence and *χ*^2^-divergence measure the discrepancies between optimal importance functions and proposed importance functions. Compared with Lemma 1, both the divergences are related to the dimensionality of the critical variables, instead of all variables, which resolves the challenge of high dimensionality for rare event estimation problem.

## Supplementary information

Supplementary Information

Peer Review File

Description of Additional Supplementary Files

Supplementary Movie 1

Supplementary Movie 2

Supplementary Movie 3

## Data Availability

The raw datasets that we used for modeling the naturalistic driving environment come from the Safety Pilot Model Deployment (SPMD) program^24^ and the Integrated Vehicle Based Safety System (IVBSS)^25^ at the University of Michigan, Ann Arbor. The processed data (e.g., empirical distributions of vehicle maneuvers) and other data that support the findings of this study are available from the corresponding author on reasonable request. Source data for figures are provided with this paper in the Supplementary Data. Source data are provided with this paper.

## Source data

Source Data

## Code availability

The highway traffic simulator and the autonomous vehicle simulation platform are publicly available, as described in the text and the relevant references^6,23^. The codes used for the generation of the naturalistic and adversarial driving environment are available from the corresponding author on reasonable request. Moreover, we have also provided the simulation architecture of our approach in Supplementary Fig. 1 and the algorithm flowchart in Supplementary Fig. 2.
