# Supplementary material for: Intelligent driving intelligence test for autonomous vehicles with naturalistic and adversarial environment
Source: Nat Commun. 2021 Feb 2;12:748. doi: 10.1038/s41467-021-21007-8 (PMC7854639; doi:10.1038/s41467-021-21007-8)
Supplement: Supplementary file 1 — Supplementary Information [file 41467_2021_21007_MOESM1_ESM.pdf]

Supplementary Information

Title
Intelligent Driving Intelligence Test for Autonomous Vehicles with Naturalistic and Adversarial Environment

Authors
Shuo Feng[1], Xintao Yan[1], Haowei Sun[1], Yiheng Feng[2], Henry X. Liu[1, 2,*]

Affiliations
[1]Department of Civil and Environmental Engineering, University of Michigan, Ann Arbor, MI, USA.
[2]University of Michigan Transportation Research Institute, Ann Arbor, MI, USA.
[*]Corresponding Author, henryliu@umich.edu

Supplemenraty Methods

Construction of Surrogate Models

This section describes the details of the surrogate models (SMs) used in this paper. Specifically, we constructed the SMs based on IDM car-following model[30] and MOBIL lane-changing model[34]. The IDM model considers the impact of both the desired speed and the desired bumper-to-bumper range on the longitudinal behavior:

$$a(t) = a_{\max}\left[1 - \left(\frac{v(t)}{v_0}\right)^{\delta} - \left(\frac{s^{\star}(t)}{s(t)}\right)^2\right], \tag{1}$$

where $a_{\max}$ is the maximum acceleration (2.0 m s^{-2}), v_0 is the desired speed (35 m s^{-1}), $v(t)$ and $s(t)$ are the velocity and the bumper-to-bumper range at the current time step t, δ is an exponent parameter (4.0), and $s^{\star}$ is the desired bumper-to-bumper range as

$$s^{\star} = s_0 + \max\left(0, v(t)\cdot T - \frac{v(t)\cdot \Delta v(t)}{2\sqrt{a_{\max}\cdot a_{\text{comf}}}}\right), \tag{2}$$

where s_0 is the minimum range at standstill (5.0 m), T is the desired time headway (1.5 s), $\Delta v(t)$ is the speed difference, and a_{comf} is the comfortable deceleration (4.0 m s^{-2}).

The MOBIL model calculates the utility of a lane-changing maneuver as

$$u = \tilde{a}_{\text{ego}} - a_{\text{ego}} + p\cdot\left(\left(\tilde{a}_{\text{new}} - a_{\text{new}}\right) + \left(\tilde{a}_{\text{old}} - a_{\text{old}}\right)\right), \tag{3}$$

where p is the politeness factor (0.1), $a_{\text{ego}}, a_{\text{new}}, a_{\text{old}}$ denote the original accelerations of the ego vehicle, the new follower in the target lane, and the old follower in the current lane, and $\tilde{a}_{\text{ego}}, \tilde{a}_{\text{new}}, \tilde{a}_{\text{old}}$ denote the new accelerations if the ego vehicle changes its lane. To capture the randomness of AVs, we modified the model as a probabilistic lane-changing model. First, the total lane change probability (p_{LC}) is calculated by using a piecewise linear function of the total utility ($u_T = \max\{0, u_L\} + \max\{0, u_R\}$):

$$p_{\mathrm{LC}} = \begin{cases} 0.9, & u_T \ge 1 \\ \left(0.9 - 2\times 10^{-8}\right) u_T, & u_T \in (0,1). \\ 2\times 10^{-8}, & u_T = 0 \end{cases} \quad (4)$$

where 2×10^{-8} indicates the minimum probability of lane change from NDD.

Then, the specific probability for the left and right lane-changing maneuver is proportionally allocated according to its utility u_L and u_R.

Construction of AV Models

This section describes the details of the two AV models used in this paper. The AV-I model was constructed by the IDM and MOBIL models with the calibrated parameters: $a_{\max}$ is 1.5 m s^{-2}, v_0 is 33.33 m s^{-1}, δ is 4, s_0 is 2.0, T is 1.2 s, a_{comf} is 2 m s^{-2}, and p is 0.0. The AV-II model was trained by DRL techniques with the efficiency reward mapping the velocity from [20, 40] (m s^{-1}) to the reward [-1, 1]. The inputs of the network were the states (velocity, position, and lane ID) of the vehicles within the AV's observation range (120 m). The outputs were the 33-dimensional discretized maneuver space, including left and right lane changes, and the 31 discrete longitudinal accelerations from -4 to 2 (m s^{-2}) with a resolution of 0.2 (m s^{-2}). The AV agent predicted behaviors of surrounding vehicles for one time step and avoided the maneuvers that lead to immediate accidents. We implemented the double deep Q-networks (DDQN) algorithm[40] with dueling networks[41] to train the AV agent. Besides the dueling layer, we used two hidden layers with 256 neurons. We set the learning rate (10^{-5}), batch size (64), discount factor (0.99), target network update frequency (1,000), replay memory size (50,000), loss function (mean square error), and the optimizer (Adam), as commonly used in the practices.

Proof of Theorem 1

By the importance sampling theory, the estimation variance can be derived as

$$\sigma^2 = E_{q(\mathbf{x})}\left(P^2\left(A|\mathbf{x}\right)\frac{P^2\left(\mathbf{x}\right)}{q^2\left(\mathbf{x}\right)}\right) - P^2\left(A\right), \quad (5)$$

where $q(\mathbf{x}) = P(\mathbf{x}_{-c})q(\mathbf{x}_c)$ denotes that the critical variables are sampled from the importance function $\mathbf{x}_c \sim q(\mathbf{x}_c)$, while other variables are sampled from the naturalistic distributions $\mathbf{x}_{-c} \sim P(\mathbf{x}_{-c})$. Substituting $q(\mathbf{x})$ into the equation, we have the following equation as

$$\begin{aligned} \sigma^2 &= E_{q(\mathbf{x})}\left(P^2\left(A|\mathbf{x}\right)\frac{P^2\left(\mathbf{x}_c\right)}{q^2\left(\mathbf{x}_c\right)}\right) - P^2\left(A\right), \\ &= E_{q(\mathbf{x}_c)}\left(E_{P(\mathbf{x}_{-c})}\left(P^2\left(A|\mathbf{x}\right)\right)\frac{P^2\left(\mathbf{x}_c\right)}{q^2\left(\mathbf{x}_c\right)}\right) - P^2\left(A\right). \end{aligned} \quad (6)$$

Substituting the variance equation that

$$E_{P(\mathbf{x}_{-c})}\left(P^2\left(A|\mathbf{x}\right)\right) = E^2_{P(\mathbf{x}_{-c})}\left(P\left(A|\mathbf{x}\right)\right) + \sigma^2_{P(\mathbf{x}_{-c})}\left(P\left(A|\mathbf{x}\right)\right), \quad (7)$$

the estimation variance equation can be further derived as

$$
\begin{aligned}
\sigma^2 &= E_{q(\mathbf{x})}\left(P^2\left(A|\mathbf{x}\right)\frac{P^2\left(\mathbf{x}_c\right)}{q^2\left(\mathbf{x}_c\right)}\right)-P^2\left(A\right), \\
&= E_{q(\mathbf{x}_c)}\left(E^2_{P(\mathbf{x}_{-c})}\left(P\left(A|\mathbf{x}\right)\right)\frac{P^2\left(\mathbf{x}_c\right)}{q^2\left(\mathbf{x}_c\right)}\right)+E_{q(\mathbf{x}_c)}\left(\sigma^2_{P(\mathbf{x}_{-c})}\left(P\left(A|\mathbf{x}\right)\right)\frac{P^2\left(\mathbf{x}_c\right)}{q^2\left(\mathbf{x}_c\right)}\right)-P^2\left(A\right).
\end{aligned} \tag{8}
$$

The first term can be derived as

$$
E_{q(\mathbf{x}_c)}\left(E^2_{P(\mathbf{x}_{-c})}\left(P\left(A|\mathbf{x}\right)\right)\frac{P^2\left(\mathbf{x}_c\right)}{q^2\left(\mathbf{x}_c\right)}\right)=E_{q(\mathbf{x}_c)}\left(P^2\left(A|\mathbf{x}_c\right)\frac{P^2\left(\mathbf{x}_c\right)}{q^2\left(\mathbf{x}_c\right)}\right). \tag{9}
$$

By the importance sampling theory, the optimal importance function for the critical variables is

$$
q^*\left(\mathbf{x}_c\right)=\frac{P\left(A|\mathbf{x}_c\right)P\left(\mathbf{x}_c\right)}{P\left(A\right)}. \tag{10}
$$

Substituting it into the equation, the first term can be further derived as

$$
\begin{aligned}
E_{q(\mathbf{x}_c)}\left(P^2\left(A|\mathbf{x}_c\right)\frac{P^2\left(\mathbf{x}_c\right)}{q^2\left(\mathbf{x}_c\right)}\right) &= P^2\left(A\right)E_{q^*(\mathbf{x}_c)}\left(\frac{q^*\left(\mathbf{x}_c\right)}{q\left(\mathbf{x}_c\right)}\right), \\
&= P^2\left(A\right)E_{q^*(\mathbf{x}_c)}\left(\exp\log\frac{q^*\left(\mathbf{x}_c\right)}{q\left(\mathbf{x}_c\right)}\right), \\
&\geq P^2\left(A\right)\exp E_{q^*(\mathbf{x}_c)}\left(\log\frac{q^*\left(\mathbf{x}_c\right)}{q\left(\mathbf{x}_c\right)}\right), \\
&= P^2\left(A\right)\left\{\exp\left[D_{\mathrm{KL}}\left(q^*(\mathbf{x}_c)\,||\,q(\mathbf{x}_c)\right)\right]-1\right\}.
\end{aligned} \tag{11}
$$

Moreover, according to the definition of χ^2-divergence, we can also derive the equivalence as

$$
E_{q^*(\mathbf{x}_c)}\left(\frac{q^*\left(\mathbf{x}_c\right)}{q\left(\mathbf{x}_c\right)}\right)=E_{q(\mathbf{x}_c)}\left(\left(\frac{q^*\left(\mathbf{x}_c\right)}{q\left(\mathbf{x}_c\right)}-1\right)^2\right)+1=D_{\chi^2}\left(q^*(\mathbf{x}_c)\,||\,q(\mathbf{x}_c)\right)+1. \tag{12}
$$

The second term can be derived as

$$
E_{q(\mathbf{x}_c)}\left(\sigma^2_{P(\mathbf{x}_{-c})}\left(P\left(A|\mathbf{x}\right)\right)\frac{P^2\left(\mathbf{x}_c\right)}{q^2\left(\mathbf{x}_c\right)}\right)=E_{q(\mathbf{x})}\left[\left(P(A\,|\,\mathbf{x})-P(A\,|\,\mathbf{x}_c)\right)^2\frac{P^2(\mathbf{x})}{q^2(\mathbf{x})}\right]. \tag{13}
$$

Substituting both the first and second terms, we can conclude the theorem.

Supplemenraty Figures

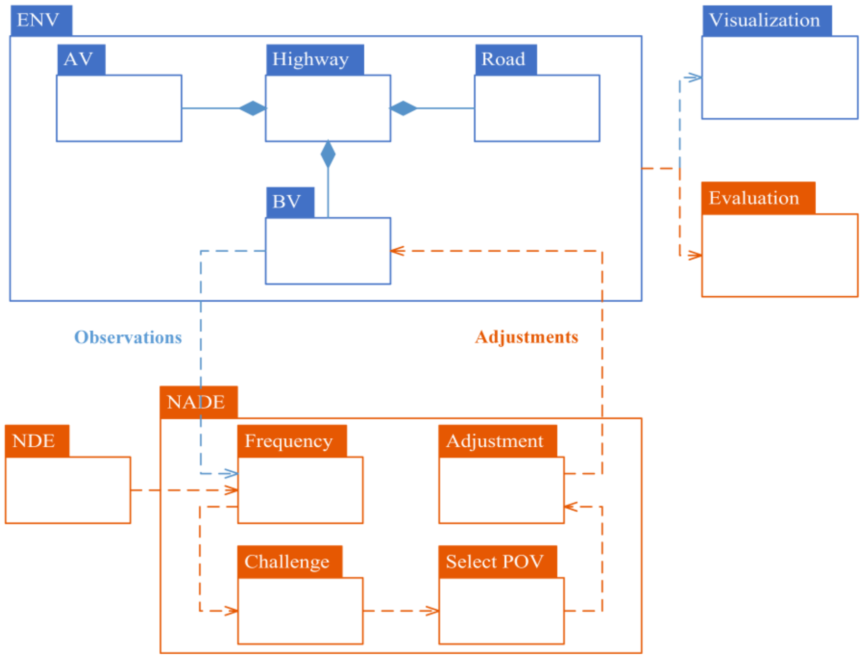

Supplementary Figure 1. Overview of the simulation architecture. We adopted the unified modeling language in this figure, where blue color denotes existing tools that are publicly available, and orange color denotes the newly developed components. The ENV acts as a highway simulator including roads, autonomous vehicle (AV), and background vehicles (BVs). At each time step, the observations of BVs are transmitted to the NADE component. Based on the empirical distributions calculated in NDE, the exposure frequency values and maneuver challenge values are calculated, and the principal other vehicle (POV) is identified if any. Then, the maneuvers are determined for all BVs, including POV and others. The simulation continues until all simulation time steps are completed or accidents happen. Finally, the testing results are visualized and analyzed.

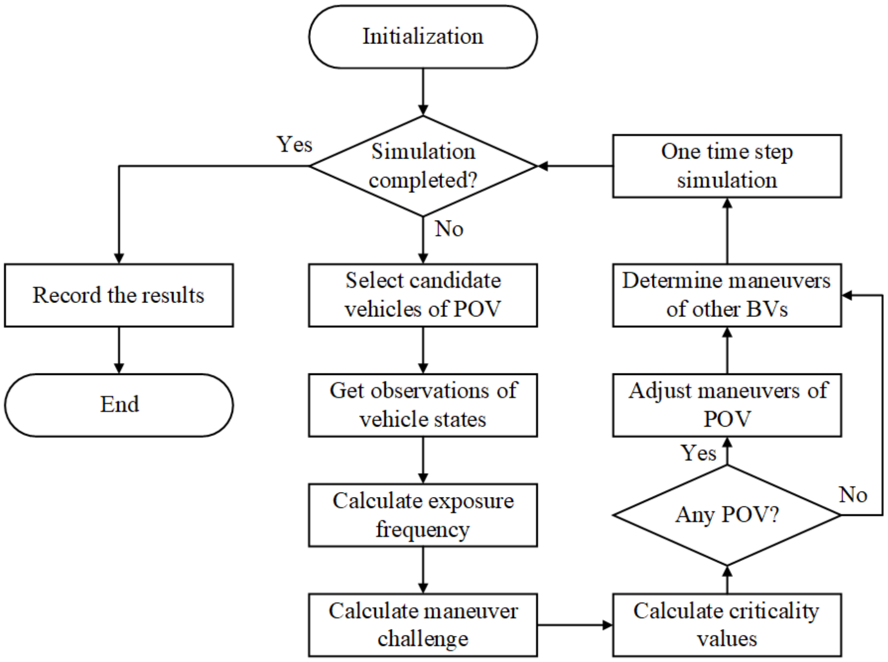

Supplementary Figure 2. Algorithm flowchart of the testing process in NADE. At each time step, the neighbor background vehicles (BVs) of the autonomous vehicle (AV) are selected as candidate vehicles of the principal other vehicle (POV). For each candidate vehicle, its exposure frequency and maneuver challenge are calculated based on its observations for the calculation of criticality values. According to the criticality values, the POV is identified inside the candidate vehicles. The maneuver of POV is adjusted by the proposed method, and other BVs are controlled following their naturalistic behaviors. The simulation continues until all simulation time steps are completed or accidents happen. After the end of one simulation, the testing results are recorded, and another simulation will begin.
