# Supplementary material for: Intelligent driving intelligence test for autonomous vehicles with naturalistic and adversarial environment
Source: Nat Commun. 2021 Feb 2;12:748. doi: 10.1038/s41467-021-21007-8 (PMC7854639; doi:10.1038/s41467-021-21007-8)
Supplement: Supplementary file 3 — Description of Additional Supplementary Files [file 41467_2021_21007_MOESM3_ESM.pdf]

Description of Additional Supplementary Files

Supplementary Movie 1: Generation of naturalistic driving environment.

Supplementary Movie 2: Generation of naturalistic and adversarial driving environment.

Supplementary Movie 3: Adversarial examples.
