# Supplementary material for: Intelligent driving intelligence test for autonomous vehicles with naturalistic and adversarial environment
Source: Nat Commun. 2021 Feb 2;12:748. doi: 10.1038/s41467-021-21007-8 (PMC7854639; doi:10.1038/s41467-021-21007-8)
Supplement: Supplementary file 2 — Peer Review File [file 41467_2021_21007_MOESM2_ESM.pdf]

Reviewer #1 (Remarks to the Author):

Overall, the research is strong, clear, interesting and is of a high standard. It posits an interesting approach to understanding some of the key challenges to autonomous vehicles and assessing safety. My first and second reading highlighted many difficulties with the overall approach and aims of the paper but with the third reading I found myself appreciating the value of the papers contribution not in achieving its interesting aim but rather in its informative analysis of a problem. Most importantly, it offers a quality resource to AV researchers and includes many interesting engaging questions and pointers to other literature in this area.

1. The Hypothetical Bottleneck

"Waymo has only simulated 63 billion miles in total over the years, which is the world's longest simulation test. To a certain extent, this inefficiency issue has become a bottleneck and has severely hindered the progress of the AV development and deployment."

Whether the framed inefficiency issue has become a fundamental bottleneck severely hindering progress is uncertain. Perhaps, the problem is intentionally amplified to give greater purpose to the paper? In places I find the tone of the paper to be to grandiose and over reaching in its claims. While it is important to sell ones research it is also important not to over sell it and I felt there was some concern here as this seemed apparent in a few places.

2. The Philosophy and supporting Grandiose claims of the paper
"They are far from representing the full complexity and variability of the real-world driving environment. For example, an AV driving in a highway-driving environment can involve various maneuvers (e.g., lane-changing, car-following, over- taking, etc.) of hundreds of vehicles for hours of time duration. None of the existing methods can be applied to generate such a driving environment, which is critical for AV testing. We make the methodological breakthrough for the generation of generic AV test environment with full complexity. Our method can dramatically reduce the simulation time with multiple orders of magnitude, and, therefore, significantly accelerate the development and deployment of AVs."

I have difficulties with the overall 'philosophy' of the paper and the authors insistence to contrast intelligence tests with machine intelligence in the context of driving. This reminds me of a category error and may point to a confused foundation that undermines the contribution of the enterprise. There are many commonalities between human and machine driving, most apparent is the fact that autonomous vehicles will function on existing human designed and formed infrastructure that will be populated with both human and machine drivers. The idea of a machine intelligence test as "critical to the development and deployment of autonomous vehicles (AVs)" is problematic to me. A part of my concern relates to the framing of agency here and the agent as an intelligent actor assessed in the same manner as human driving proficiency. I also find some of the claims relating to the application to be grandiose. For example; "We demonstrate the effectiveness of NADE on two AV agents in a highway-driving scenario. Comparing with NDE, the NADE can accelerate the evaluation process by multiple orders of magnitude."

There is no doubt the research is interesting, original in scope and makes a contribution to the literature set but I find the extent of the contribution is not in what it claims in terms of accelerating AV "evaluation process by multiple orders of magnitude" but rather in the interesting content and material within the research. This is why I think reconsideration of the framing of the paper may be helpful.

3. The Framing of human and machine driving Intelligence tests:
While the authors point to their unfamiliarity with intelligence testing and theory, and the questionable

aspects to applying this to machine intelligence as an effective assessment, they still hold to this inference as an important one for their approach. I think section 36-45 needs fleshing out more to support the aims of the paper. Human intelligence tests have been utilised and developed for decades and much of the theory and application has come under scrutiny. So much so, that some now suggest that a great deal of dogmatism relating to theory and credibility of human intelligence tests defined much of the inferences and logic to them (see). My point is that human intelligence tests (for me and others) are problematic for many reasons and forwarding a position founded on the success and applicability of human intelligence test (theory) to support the development of a test for machines is problematic. There is a leap of faith that the paradigm of human intelligence tests (even if it was a successful approach) can sufficiently apply to machine driving intelligence. I don't believe it is a good strategy. That said, there is a logic to it that us worth further consideration and research. Given that this is only one aspect to the paper (an important one framing the paper's contribution) and there is a great deal more that is worthy in the paper in terms of the potential application of NADE, I believe that with minor considerations and a reappraisal of the overall framing the paper is worthy of publication in this journal. I must emphasise that I do not believe that the use of NADE to support machine intelligence for real world driving can provide a magic bullet solution to artificial driving intelligence and safety proficiency. Rather, the paper should develop and focus on the potential supporting applications of NADE in contributing to developing machine solutions to difficult real world driving scenarios. Simulators are commonly used and there is often a focus on complex decision and pre-accident events.

(Perhaps, the above critical response is too philosophical and displays my own bias relating to my dislike of the approach. The paper, the overall approach and the wealth of information relating to AV research further supports the strength of the paper as making a contribution to the literature set relating to autonomous vehicles. With this is mind I feel that the research field would certainly benefit from the paper. Moreover, the contribution may well be greater if I am wrong in terms of my views regarding the category error).

4. Coding variables and simulating random driving behaviour
I think this is a promising aspect of the paper but needs more development in terms of verifying its value.
"The resulting driving environment is both naturalistic and adversarial, in that most of the background vehicles (more generally, road users) follow naturalistic behaviors for most of the time, and only at selected moments, selected vehicles execute specific designed adversarial moves."

Again, this maybe too philosophical but I keep coming back to what exactly the machine learning behaviour algorithm is actually learning. Is it simply a complex optimisation algorithm concerning object avoidance but made more complex by framing it in human behavioural language.

I am unsure about the legitimacy of this key supporting claim in terms of it at least requires further support.
"For safety-critical intelligence test of AVs, fortunately, these small but critical variables usually exist because most of the vehicle accidents involve only a small number of vehicles in a short period (22). This discovery provides a solid theoretical foundation for our methodological breakthrough."

5. The application and value of the NADE framework

Of course the NADE framework can be applied to any machine decision context as it simulates the dynamic and changing values by introducing variables and relations and this is sterilised in a simulated format to support machine intelligence to quantify the scenario to learn optimum outputs. This method has benefits but at most can only be part of an overall more complex testing/learning approach/methodology. This is because on its own the framework cannot suffice and the overall benefit and results offered will remain uncertain and questionable. It was helpful to see the authors

identify some of the limitations of the framework.

Final recommendation:

I come back to my original intuition that the research clearly is of a high standard and makes a positive contribution to AV literature in several ways. This remains the case, even if I find difficulties with the overall philosophy, framing and conceptualisation of machine decisionality. With this in mind I recommend it deserves publication in this journal.

Reviewer #2 (Remarks to the Author):

This is a well written and thought provoking paper on an important open problem in autonomous driving. Specifically, testing the effectiveness of autonomous vehicle (AV) in simulation requires millions or even billions of miles of runs because of the nature of rare events that might cause the accidents. Authors propose instead adversarial environment generation (i.e. NADE) that can accelerate finding edge cases by few orders of magnitude.

The authors have been inspired by the approach in AlphaGo however the approach they describe is not the same. On high level, the driving can be seen as decision tree unfolding much like a game of Go where each edge is action taken by the AV while each node is state of the vehicles. The main idea is to use some method (here IS + RL) to look ahead to increase the probability of finding the node that will be an accident. As opposed to crude Monte Carlo, this will obviously accelerate finding the edge cases. In some respect this might be similar to what if surrounding vehicles were adversarial to one driver to cause them to collide within certain allowed distribution of actions. One of the most attractive aspect of the presented approach is that the distribution within NADE is very close the NDE environment which in part also leads of statistical unbiasedness.

Authors take here methodical approach utilizing importance sampling and reinforcement learning to generate adversarial behavior. A theorem and a proof is also provided for the unbiasedness for the performance evaluation.

My primary concerns are as follows:

1. While the method presented in the paper could be unbiased statistically, it still does not suggest that the *type* of accidents detected by this method is same as the ones that will be found in NDE. My concern is that the type of accidents found in NDE could be different vs the accidents caused by adversarial behavior. To properly address this concern, accident state sequences should be recorded in NDE and compared with ones in NADE to compute the intersection between two sets and use some set similarity measure.

2. The "adversarialness" generated by this method seems limited and not truly learned, despite of RL, as it depends on rather arbitrary hand crafted measures of "criticality". The primary concern here is that next AV update can be trained against this less robust "adversarialness" and suddenly show a significant jump in test by being adversarial itself. For truly learned approach, better algorithms are needed instead of simple IS with criticality measure.

3. The paper has a long series of simplifying assumptions (only highway driving, only few actions, only one POV etc) that limits the immediate utility for practical applications however in author's defence, their method does seem scalable to more complex scenario. However it is not clear if various parameters (specifically e) and just selecting one POV could be sufficient and if RL can still be effective

when action space is increased dramatically.

4. A significant value could have been generated if authors could demonstrate that the *type* of accidents that occur in real world are in fact detected by their method as opposed to only finding possibly extreme rare case scenarios that might be just outcome of adversarial behavior. This however may require acquiring accident dataset and some manual annotation work. Still, if there is a proof that none of the top N accidents types are missed for detection by this approach, it can generate far more confidence is practical utility of this method.

5. It would be very useful to readers if author can also include pseudocode format and/or architecture diagram that shows all components.

6. Nit pick: A possible missing word in line 454 "such as those *from*".

7. I did not saw any mention of code release. Given the number of moving parts and missing implementation details, I expect results won't be reproducible without significant investment, if at all. However, ideas are easily understood and can be built upon.

8. I should emphasis that all experiments and evaluations are in simulation and no real world tests have been conducted of any kind.

Overall, the paper presents and original and thought provoking approach. While I suspect in its current form it might have very limited utility for the reasons mentioned above, I feel optimistic that it can seed some great future work in this important problem space. Given this, I would recommend Weak Accept for this paper.

Reviewer #3 (Remarks to the Author):

The paper presents a paradigm for testing AI-powered autonomous vehicles (AV) using adversarial examples for the driving behaviors of the background vehicles (BV) in a highway driving scenario. The initial behaviors of the background vehicles are first modeled from real-world highway scenes using standard object and lane detection techniques. In a subsequent step, these are re-modeled using an adversarial examples based on surrogate (I guess deep learning) models.

My main concerns with this paper are:
- lack of novelty. The methodology described in the paper has been already presented in papers describing adversarial networks or modeling of the driving scene's dynamics (e.g. [1]).
- the driving model is overly simplistic. The main challenge with AVs today is driving in complex and cluttered environments, such as cities or crowded streets. However, the paper presents just a simulated highway driving, without even considering different actors (all BVs are the same; modeling trucks or motorcycles would help). Autonomous driving is mostly solved for the case of highway driving.
- the authors mention that a lane change is detected based on a threshold distance from the ego-vehicle to the lane markings (see Fig. 2). This is quite a poor choice, since it may not be applicable at all to the actual dynamics of a lane change. Typically some form of temporal dynamics learning should be involved.
- in a paper describing testing of autonomous vehicles, there is not a single paragraph mentioning how the proposed system maps to functional safety standards such as the ISO 26262 or the ASIL levels.
- simulated environments tend to be highly biased towards their real-world counterparts. The paper presents a theoretical proof that the proposed system is unbiased. However, this claim is not proven in any real-world experiments.
- in this reviewer's opinion, the key to self-driving cars (both implementation and testing) lies in overcoming the data bottleneck. We simply cannot expect to train these systems solely by throwing

large quantities of data to them.

I do hope that my comments will help in improving your work.
I also apologize if I was too straightforward with my remarks.

[1] Mikael Henaff, Alfredo Canziani, Yann LeCun, MODEL-PREDICTIVE POLICY LEARNING WITH UNCERTAINTY REGULARIZATION FOR DRIVING IN DENSE TRAFFIC, ICRL 2019.

Best regards,
Prof. Sorin Grigorescu
ROVIS Lab (Robotics, Vision and Control Laboratory)
http://rovislab.com/sorin_grigorescu.html

Response to the Reviewers' Comments

The authors would like to thank the editor and reviewers for their constructive comments. In response to these comments, we have revised the paper carefully. We believe the revised paper has adequately addressed the issues raised in the reviewers' comments. In the following, we will provide a point-to-point response to all comments. Major revisions to the original submission are highlighted in red in the revised manuscript.

Reviewer 1

Overall, the research is strong, clear, interesting and is of a high standard. It posits an interesting approach to understanding some of the key challenges to autonomous vehicles and assessing safety. My first and second reading highlighted many difficulties with the overall approach and aims of the paper but with the third reading I found myself appreciating the value of the papers contribution not in achieving its interesting aim but rather in its informative analysis of a problem. Most importantly, it offers a quality resource to AV researchers and includes many interesting engaging questions and pointers to other literature in this area.

Response:

We thank the reviewer for the positive evaluation of the work.

1. *The Hypothetical Bottleneck*

"Waymo has only simulated 15 billion miles in total over the years, which is the world's longest simulation test. To a certain extent, this inefficiency issue has become a bottleneck and has severely hindered the progress of the AV development and deployment."

Whether the framed inefficiency issue has become a fundamental bottleneck severely hindering progress is uncertain. Perhaps, the problem is intentionally amplified to give greater purpose to the paper? In places I find the tone of the paper to be to grandiose and over reaching in its claims. While it is important to sell ones research it is also important not to over sell it and I felt there was some concern here as this seemed apparent in a few places.

Response:

We agree with the reviewer and accept the criticism that, in various places in the paper, the tone is grandiose and overreaching. It is certainly not intentional. We have rewritten the Introduction section of the paper and removed the words that may make the paper sound grandiose. Specifically, we have changed the sentence "To a certain

extent, this inefficiency issue has become a bottleneck and has severely hindered the progress of the AV development and deployment." To "To certain extent, this inefficiency issue has hindered the progress of the AV development and deployment."

2. *Philosophy and supporting Grandiose claims of the paper*

"They are far from representing the full complexity and variability of the real-world driving environment. For example, an AV driving in a highway-driving environment can involve various maneuvers (e.g., lane-changing, car-following, over- taking, etc.) of hundreds of vehicles for hours of time duration. None of the existing methods can be applied to generate such a driving environment, which is critical for AV testing. We make the methodological breakthrough for the generation of generic AV test environment with full complexity. Our method can dramatically reduce the simulation time with multiple orders of magnitude, and, therefore, significantly accelerate the development and deployment of AVs."

I have difficulties with the overall 'philosophy' of the paper and the authors insistence to contrast intelligence tests with machine intelligence in the context of driving. This reminds me of a category error and may point to a confused foundation that undermines the contribution of the enterprise. There are many commonalities between human and machine driving, most apparent is the fact that autonomous vehicles will function on existing human designed and formed infrastructure that will be populated with both human and machine drivers. The idea of a machine intelligence test as "critical to the development and deployment of autonomous vehicles (AVs)" is problematic to me. A part of my concern relates to the framing of agency here and the agent as an intelligent actor assessed in the same manner as human driving proficiency. I also find some of the claims relating to the application to be grandiose. For example; "We demonstrate the effectiveness of NADE on two AV agents in a highway-driving scenario. Comparing with NDE, the NADE can accelerate the evaluation process by multiple orders of magnitude."

There is no doubt the research is interesting, original in scope and makes a contribution to the literature set but I find the extent of the contribution is not in what it claims in terms of accelerating AV "evaluation process by multiple orders of magnitude" but rather in the interesting content and material within the research. This is why I think reconsideration of the framing of the paper may be helpful.

Response:

We have reframed the paper and rewritten the Introduction section. We agree that starting from human intelligence test in the original version is confusing and unnecessary. To address this issue, in the revised paper, we have highlighted the importance and challenges of the autonomous vehicle (AV) testing problem, discussed the limitations of state-of-the-art approaches, and summarized the contributions of our

study. Please see the Introduction section in the revised paper.

We also agree with the reviewer’s comment about the contribution of the paper. In the revised paper, we have summarized three contributions on our approach: First, our approach generates driving environment that provides spatiotemporally continuous testing scenarios for AVs, which cannot be handled by existing scenario-based approaches. Suppose you want to test an AV in an urban environment, our approach can drive the AV continuously for miles during one test, interacting with multiple background vehicles and experiencing different adversarial maneuvers. Second, the generated environment provides statistically accurate testing results. Our approach ensures that the testing results (such as accident rates of different accident types) of AVs in the generated environment are unbiased with the NDE. Third, the generated environment addresses the inefficiency issue of the NDE. Please see the Introduction section in the revised paper.

We have added the above explanation in the Introduction section in the revised paper.

3. *The Framing of human and machine driving Intelligence tests:*

While the authors point to their unfamiliarity with intelligence testing and theory, and the questionable aspects to applying this to machine intelligence as an effective assessment, they still hold to this inference as an important one for their approach. I think section 36-45 needs fleshing out more to support the aims of the paper. Human intelligence tests have been utilised and developed for decades and much of the theory and application has come under scrutiny. So much so, that some now suggest that a great deal of dogmatism relating to theory and credibility of human intelligence tests defined much of the inferences and logic to them (see). My point is that human intelligence tests (for me and others) are problematic for many reasons and forwarding a position founded on the success and applicability of human intelligence test (theory) to support the development of a test for machines is problematic. There is a leap of faith that the paradigm of human intelligence tests (even if it was a successful approach) can sufficiently apply to machine driving intelligence. I don't believe it is a good strategy. That said, there is a logic to it that us worth further consideration and research. Given that this is only one aspect to the paper (an important one framing the paper's contribution) and there is a great deal more that is worthy in the paper in terms of the potential application of NADE, I believe that with minor considerations and a reappraisal of the overall framing the paper is worthy of publication in this journal.

Response:

Again, we agree with the reviewer that starting from human intelligence test in the original version is confusing and unnecessary. In the revised paper, we have removed the analogy to human intelligence test or machine intelligence test. Moreover, we have

highlighted the importance and challenges of the AV testing problem, discussed the limitations of state-of-the-art approaches, and summarized the contributions of our study. Please see the Introduction section in the revised paper.

I must emphasise that I do not believe that the use of NADE to support machine intelligence for real world driving can provide a magic bullet solution to artificial driving intelligence and safety proficiency. Rather, the paper should develop and focus on the potential supporting applications of NADE in contributing to developing machine solutions to difficult real world driving scenarios. Simulators are commonly used and there is often a focus on complex decision and pre-accident events.

Response:

We agree with the reviewer that NADE is not a magic bullet solution to artificial driving intelligence and it is not the purpose of this paper as well. In the revised paper, we argue that NADE provides a methodological framework to simulate a driving environment that can provide spatiotemporally continuous testing scenarios, which is unbiased and efficient comparing with its NDE counterpart. We also agree that NADE has potential to support various applications for autonomous vehicle development, especially under difficult real world driving scenarios. For example, NADE is promising for AV training because of the two reasons: First, because difficult driving scenarios can be experienced by AVs more frequently than NDE (i.e., adversarial), the AVs have more opportunities to improve their performance for these extreme scenarios. Second, as the NADE is very close to NDE most of the time (i.e., naturalistic), it is also promising to avoid overfitting the AV agents in these extreme scenarios. We leave the applications of NADE for future work.

Perhaps, the above critical response is too philosophical and displays my own bias relating to my dislike of the approach. The paper, the overall approach and the wealth of information relating to AV research further supports the strength of the paper as making a contribution to the literature set relating to autonomous vehicles. With this is mind I feel that the research field would certainly benefit from the paper. Moreover, the contribution may well be greater if I am wrong in terms of my views regarding the category error.

Response:

Thanks for your positive feedback!

4. *Coding variables and simulating random driving behaviour*

I think this is a promising aspect of the paper but needs more development in terms

of verifying its value.

"The resulting driving environment is both naturalistic and adversarial, in that most of the background vehicles (more generally, road users) follow naturalistic behaviors for most of the time, and only at selected moments, selected vehicles execute specific designed adversarial moves."

Again, this maybe too philosophical but I keep coming back to what exactly the machine learning behaviour algorithm is actually learning. Is it simply a complex optimisation algorithm concerning object avoidance but made more complex by framing it in human behavioural language.

Response:

To answer the question about what exactly the learning algorithm is actually learning, let us look into the essence of the AV testing problem. As we discussed in the Introduction section, the driving intelligence testing of AVs is essentially a rare event estimation problem with high-dimensional variables. Specifically, as elaborated in Methods section, the goal of testing an AV is to evaluate its performance

$$P(A) = E_{x\sim P(x)}\big(P(A|x)\big),$$

where A denotes the event of interest (e.g., accident event), x denotes variables of the driving environment (e.g., behaviors of background vehicles), and $P(x)$ denotes the naturalistic distribution of x in NDE. The NDE-based testing is essentially to estimate $P(A)$ (e.g., accident rate) by the Crude Monte Carlo (CMC) method as

$$P(A) = E_{x\sim P(x)}\big(P(A|x)\big) \approx \frac{1}{n}\sum_{i=1}^{n} P(A|x_i) \approx \frac{m}{n}, x_i \sim P(x),$$

where n denotes the number of tests, and m denotes the number of event A occurred during the tests.

Because the event A is a rare event for AVs in NDE, the method requires a large n to obtain a reasonable estimation precision, which causes the inefficiency limitation of NDE-based testing. To address this limitation, the importance sampling method is applied to estimate the performance by a new distribution $q(x)$, denoted as the importance function in this paper, as

$$P(A) = E_{x\sim P(x)}\big(P(A|x)\big)$$

$$= E_{x\sim q(x)}\left(P(A|x)\frac{P(x)}{q(x)}\right)$$

$$\approx \frac{1}{n}\sum_{i=1}^{n} P(A|x_i)\frac{P(x_i)}{q(x_i)}, x_i \sim q(x).$$

According to the importance sampling theory, if $q(x)$ is "well designed" (satisfying

several requirements), the inefficiency limitation can be addressed and the unbiasedness can be maintained. However, as the high dimensionality of the driving environment, namely when x is high dimensional, constructing the well designed $q(x)$ is a challenging problem.

In this paper, we discover that sparse but adversarial adjustment of NDE ($x \sim P(x)$) can essentially result in the well-designed importance function $q(x)$, which generates the new testing environment ($x \sim q(x)$). As behaviors of all background vehicles are determined by the variables (x), ***the learning algorithm for background vehicles is actually learning when and how to adjust the behaviors of which background vehicles so that the importance function $q(x)$ can satisfy the requirement of importance sampling.*** We find that the resulting driving environment is both naturalistic and adversarial, in that most of the background vehicles follow naturalistic behaviors for most of the time, and only at selected moments, selected vehicles execute specific designed adversarial moves.

We have revised the paper accordingly. In the revised paper, please see the sixth and seventh paragraphs in the Introduction section and the Methods section for more details on how to construct the importance function.

I am unsure about the legitimacy of this key supporting claim in terms of it at least requires further support.

"For safety-critical intelligence test of AVs, fortunately, these small but critical variables usually exist because most of the vehicle accidents involve only a small number of vehicles in a short period (22). This discovery provides a solid theoretical foundation for our methodological breakthrough."

Response:

We have revised these sentences to avoid the possible confusion. The key supporting claim here is that, if there exists a small subset of variables that are critical to the rare events, applying the importance sampling (IS) theory with the small subset of variables while applying the Crude Monte Carlo (CMC) theory with the remaining variables can help overcome both the challenges of the rareness of events and high dimensionality. To justify the claim, we provide a theoretical proof of this in Theorem 1 in Methods section.

For safety-critical robotics, comparing with the high dimensional variables that define the driving environment, there usually exist a subset of variables that are more important for safety-critical events than others are. According to the Fatality Analysis Reporting System (FARS), about 91.5% fatal injuries suffered in motor vehicle traffic

crashes at the United State in 2018 involved only one or two vehicles[1]. Therefore, in NADE, we only need to adjust the behavior of the critical background vehicles for a short period of time, while maintain all other background vehicles with naturalistic driving behavior. This will satisfy the requirements for importance sampling.

We have revised the paper accordingly. Please see the sixth and seventh paragraphs in the Introduction section in the revised paper.

5. *The application and value of the NADE framework*

Of course the NADE framework can be applied to any machine decision context as it simulates the dynamic and changing values by introducing variables and relations and this is sterilised in a simulated format to support machine intelligence to quantify the scenario to learn optimum outputs. This method has benefits but at most can only be part of an overall more complex testing/learning approach/methodology. This is because on its own the framework cannot suffice and the overall benefit and results offered will remain uncertain and questionable. It was helpful to see the authors identify some of the limitations of the framework.

Response:

We have revised the Discussion section to discuss the limitations of the framework, including approximation error of maneuver challenge, data requirement, simplifications of case study, and lack of perception related tests. We agree that further development and enhancement are required for improving the practical utility of our approach. Please see the Discussion section in the revised paper.

Final recommendation:

I come back to my original intuition that the research clearly is of a high standard and makes a positive contribution to AV literature in several ways. This remains the case, even if I find difficulties with the overall philosophy, framing and conceptualisation of machine decisionality. With this in mind I recommend it deserves publication in this journal.

Response:

Thanks for your positive feedback again!

[1] The data is publicly available in https://www.nhtsa.gov/content/nhtsa-ftp/176776.

Reviewer 2

This is a well written and thought provoking paper on an important open problem in autonomous driving. Specifically, testing the effectiveness of autonomous vehicle (AV) in simulation requires millions or even billions of miles of runs because of the nature of rare events that might cause the accidents. Authors propose instead adversarial environment generation (i.e. NADE) that can accelerate finding edge cases by few orders of magnitude.

The authors have been inspired by the approach in AlphaGo however the approach they describe is not the same. On high level, the driving can be seen as decision tree unfolding much like a game of Go where each edge is action taken by the AV while each node is state of the vehicles. The main idea is to use some method (here IS + RL) to look ahead to increase the probability of finding the node that will be an accident. As opposed to crude Monte Carlo, this will obviously accelerate finding the edge cases. In some respect this might be similar to what if surrounding vehicles were adversarial to one driver to cause them to collide within certain allowed distribution of actions. One of the most attractive aspect of the presented approach is that the distribution within NADE is very close the NDE environment which in part also leads of statistical unbiasedness.

Authors take here methodical approach utilizing importance sampling and reinforcement learning to generate adversarial behavior. A theorem and a proof is also provided for the unbiasedness for the performance evaluation.

Response:

Thanks for your positive feedback.

My primary concerns are as follows:

*1. While the method presented in the paper could be unbiased statistically, it still does not suggest that the *type* of accidents detected by this method is same as the ones that will be found in NDE. My concern is that the type of accidents found in NDE could be different vs the accidents caused by adversarial behavior. To properly address this concern, accident state sequences should be recorded in NDE and compared with ones in NADE to compute the intersection between two sets and use some set similarity measure.*

Response:

We have added more analysis of the testing results on the accident types in the

revised paper. We have adopted the crash type diagram[1] defined by the Fatality Analysis Reporting System (FARS)[2], which is a nationwide census provided by National Highway Traffic Safety Administration (NHTSA) on data regarding fatal injuries suffered in motor vehicle traffic crashes. For the highway driving case in this paper, we only have the accidents between the AV and BVs, so the five accident types are studied, as shown in Fig. 7f in the revised paper. We also compared the results for the AV-II model in both NDE and NADE, and demonstrated the unbiasedness of NADE in terms of accident types. This is shown in Fig. 7 (g, h) in the revised paper.

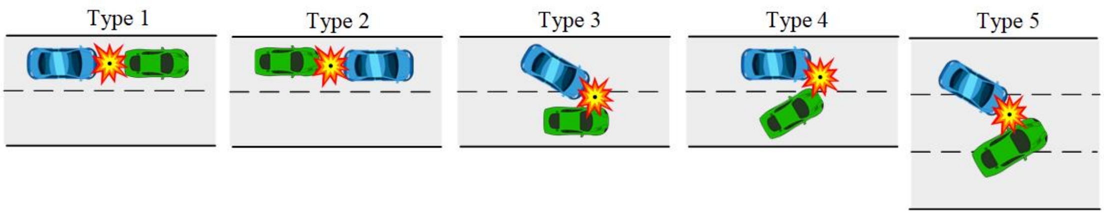

Fig. 7f. Illustrations of the accident types. Type 1: the AV has a rear-end collision with the BV. Type 2: the BV has a rear-end collision with the AV. Type 3: the AV makes a lane change and has a sideswipe collision with the BV. Type 4: the BV makes a lane change and has a sideswipe collision with the AV. Type 5: both the AV and BV make lane change and have a sideswipe collision.

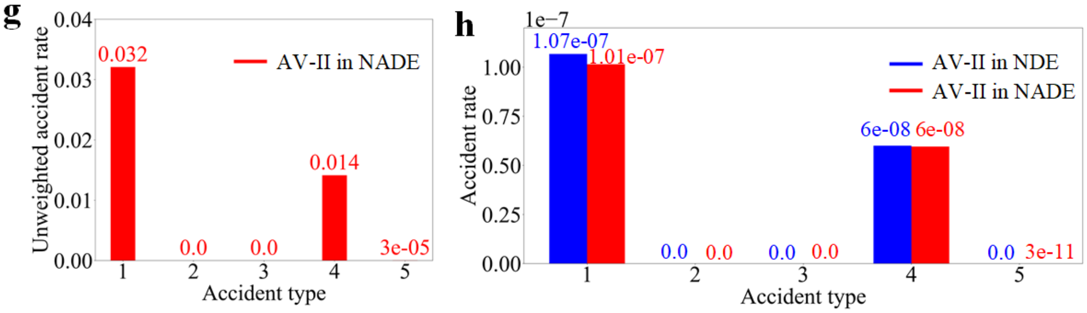

Fig. 7. g Unweighted accident rate for each type in NADE. **h** Accident rate for each type in NDE (blue) and weighted accident rate of each type in NADE (red).

The statistical unbiasedness for accident types can also be obtained by theoretical analysis. As elaborated in the Methods section, the NDE-based testing is to estimate the AV performance as

$$P(A) = E_{x\sim P(x)}\left(P(A|x)\right),$$

[1] National Center for Statistics and Analysis. (2019, September). Fatality Analysis Reporting System (FARS) Analytical User's Manual, 1975-2018 (Report No. DOT HS 812 827). Washington, DC: National Highway Traffic Safety Administration.

[2] https://www.nhtsa.gov/research-data/fatality-analysis-reporting-system-fars.

where A denotes the event of interest (e.g., accident event), x denotes variables of the driving environment (e.g., behaviors of background vehicles), and $P(x)$ denotes the naturalistic distribution of x in NDE. The NADE-based testing is essentially to evaluate the AV performance by a new driving environment ($x \sim q(x)$) as

$$E_{x\sim q(x)}\left(P(A|x)\frac{P(x)}{q(x)}\right) \approx \frac{1}{n}\sum_{i=1}^{n} P(A|x_i)\frac{P(x_i)}{q(x_i)}, x_i \sim q(x)$$

where $q(x)$ is denoted as the importance function in this paper, and $\frac{P(x_i)}{q(x_i)}$ is the likelihood ratio of each simulation test (i.e., simulation weight). Based on the importance sampling (IS) theory, the evaluation is unbiased, namely

$$E\left(\frac{1}{n}\sum_{i=1}^{n} P(A|x_i)\frac{P(x_i)}{q(x_i)}\right) = E_{x\sim q(x)}\left(P(A|x)\frac{P(x)}{q(x)}\right) = P(A),$$

if the following condition is satisfied: $q(x) > 0$ whenever $P(A|x)P(x) \neq 0$. As a defensive importance sampling strategy will be used in the generation of NADE, i.e., the naturalistic distribution will also be explored with a very small probability, we can guarantee that $q(x) > 0$ whenever $P(x) \neq 0$, which is sufficient for the unbiasedness for any event A. Therefore, for any type of accident event A, its accident rate $P(A)$ can be evaluated in a statistical unbiased way.

2. The "adversarialness" generated by this method seems limited and not truly learned, despite of RL, as it depends on rather arbitrary hand crafted measures of "criticality". The primary concern here is that next AV update can be trained against this less robust "adversarialness" and suddenly show a significant jump in test by being adversarial itself. For truly learned approach, better algorithms are needed instead of simple IS with criticality measure.

Response:

We understand the reviewer's concern on "adversarialness". The very reason we define the criticality as the product of maneuver challenge and exposure frequency is that "adversarialness" alone may lead to biased evaluation results for AV safety performance. In NADE, the goal of background vehicles is not simply to be adversarial to defeat the AV agent, but to test the AV agent in a naturalistic and adversarial manner that is consistent with importance sampling theory. Our criticality definition is inspired by the risk assessment model in ISO 26262, where the risk assessment of a scenario was defined as a combination of severity of injuries, exposure classification, and controllability classification. The exposure classification denotes the relative expected exposure frequency of the scenario where the injury can possibly happen. The controllability classification denotes the relative likelihood that the driver can act to

prevent the injury. More details on the criticality definition can be found on our previous work[3].

We also agree that the proposed RL-based method can be further improved for learning the "criticality" values, as we discussed in the Discussion section (see the second paragraph in that section). As learning the criticality values is essentially a policy evaluation in the AI domain, it is straightforward to enhance our approach by utilizing advanced AI algorithms such as those from deep reinforcement learning (DRL) with Monte Carlo Tree Searching techniques, similar to the approach in AlphaGo.

3. The paper has a long series of simplifying assumptions (only highway driving, only few actions, only one POV etc) that limits the immediate utility for practical applications however in author's defence, their method does seem scalable to more complex scenario. However it is not clear if various parameters (specifically e) and just selecting one POV could be sufficient and if RL can still be effective when action space is increased dramatically.

Response:

We agree that the case study in this paper has several simplifications for the convenience of experiments, which limits the immediate utility for practical applications. However, the theoretical analysis and methodological framework are not limited to these simplifications. As shown in the Methods section, our analysis is generic for the agent-environment framework with general environment (x), action space (u), and critical variables (x_c). Therefore, our approach can also be applied to more complex scenarios such as city driving.

The selection of POVs is essentially the identification of critical variables (x_c), which is related to the evaluation efficiency. As shown in Theorem 1, to reduce the estimation variance (σ^2), x_c should be identified to capture the major causes of the event A (i.e., to reduce $D(x_c||x)$) by as few dimension of x_c as possible (i.e., to reduce $D_{\chi^2}(q^*(x_c)||q(x_c))$). As x_c is not limited to a specific dimension, the number of POV is also not limited to one, and more POVs can be selected. The reason why we select one POV in this paper is that most of the vehicle accidents involve only a small number of vehicles. For instance, according to the Fatality Analysis Reporting System (FARS), about 91.5% fatal injuries suffered in motor vehicle traffic crashes at the United State in 2018 involved only one or two vehicles[4]. Please note, as the non-POVs

---

[3] Feng, S., Feng, Y., Sun, H., Zhang, Y. and Liu, H.X., 2020. Testing Scenario Library Generation for Connected and Automated Vehicles: An Adaptive Framework. IEEE Transactions on Intelligent Transportation Systems. DOI: 10.1109/TITS.2020.3023668.

[4] The data is publicly available in https://www.nhtsa.gov/content/nhtsa-ftp/176776.

behavior follows naturalistic driving distributions, the traffic crashes involving more vehicles still have the probability to be generated by the NADE, keeping the evaluation unbiased. For example, a three-vehicle crash can be caused by one POV and two non-POVs.

The RL may become ineffective for learning the criticality values, when the action space is increased dramatically. As discussed in the Response to the second question, advanced AI techniques such as DRL can be applied to address this issue, following the same framework proposed in this paper.

*4. A significant value could have been generated if authors could demonstrate that the *type* of accidents that occur in real world are in fact detected by their method as opposed to only finding possibly extreme rare case scenarios that might be just outcome of adversarial behavior. This however may require acquiring accident dataset and some manual annotation work. Still, if there is a proof that none of the top N accidents types are missed for detection by this approach, it can generate far more confidence is practical utility of this method.*

Response:

As discussed in our Response to the first question, we have adopted the crash type diagram defined in FARS, and analyzed the testing results on the accident types. The statistical unbiasedness of all five crash types is validated by the results and theoretical analysis. Our approach is significantly different from only finding possibly extreme rare case scenarios that might be just outcome of adversarial behaviors.

5. It would be very useful to readers if author can also include pseudocode format and/or architecture diagram that shows all components.

Response:

We have added the simulation architecture and algorithm flowchart of our approach as shown in the Supplementary Materials in the revised paper.

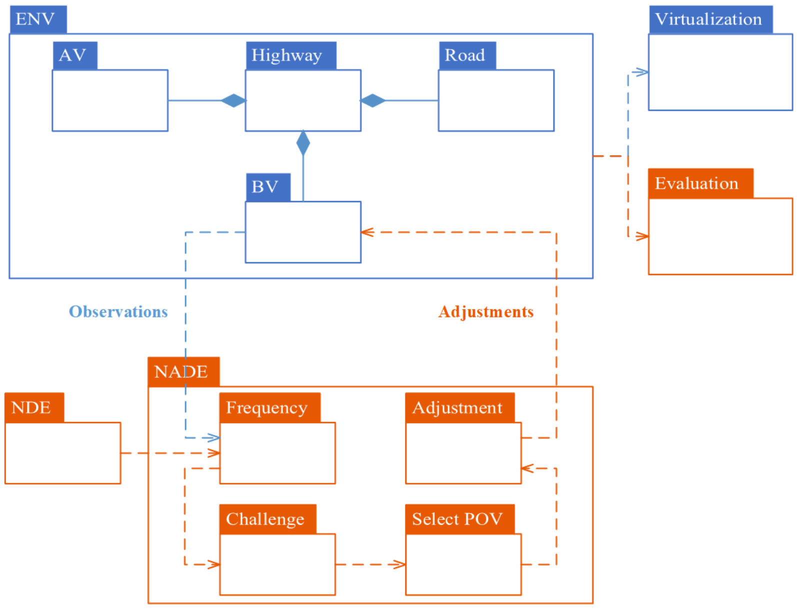

Figure S1. Overview of the simulation architecture. We adopted the unified modeling language in this figure, where blue color denotes existing tools that are publicly available, and orange color denotes the newly developed components. The ENV acts as a highway simulator including roads, autonomous vehicle (AV), and background vehicles (BVs). At each time step, the observations of BVs are transmitted to the NADE component. Based on the empirical distributions calculated in NDE, the exposure frequency values and maneuver challenge values are calculated, and the principal other vehicle (POV) is identified if any. Then, the maneuvers are determined for all BVs, including POV and others. The simulation continues until all simulation time steps are completed or accidents happen. Finally, the testing results are virtualized and analyzed.

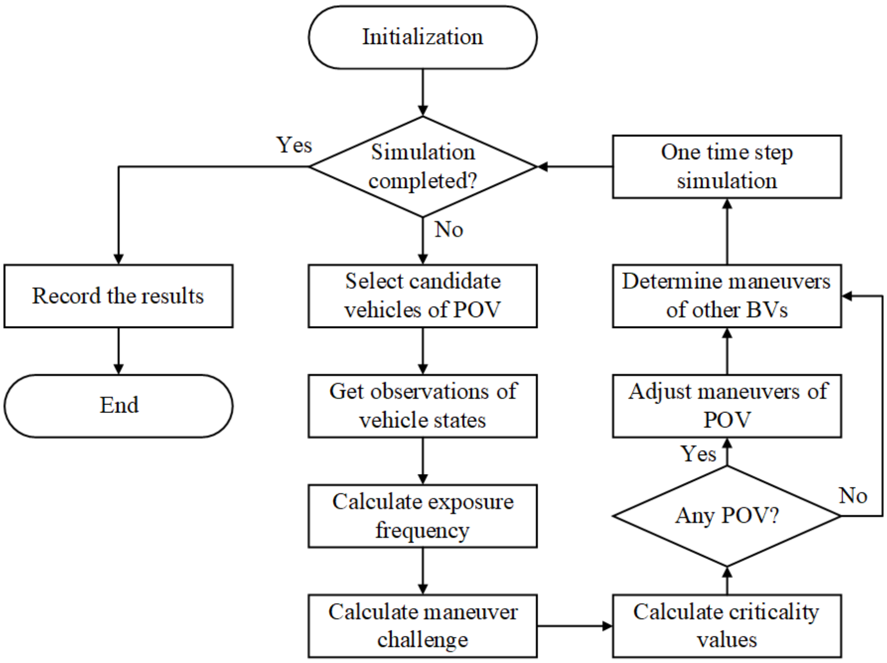

Figure S2. Algorithm flowchart of the testing process in NADE. At each time step,

the neighbor BVs of the AV are selected as candidate vehicles of POV. For each candidate vehicle, its exposure frequency and maneuver challenge are calculated based on its observations for the calculation of criticality values. According to the criticality values, the POV is identified inside the candidate vehicles. The maneuver of POV is adjusted by the proposed method, and other BVs are controlled following their naturalistic behaviors. The simulation continues until all simulation time steps are completed or accidents happen. After the end of one simulation, the testing results are recorded, and another simulation will begin.

*6. Nit pick: A possible missing word in line 454 "such as those *from*".*

Response:

We have added the missing word in the revised paper.

7. I did not saw any mention of code release. Given the number of moving parts and missing implementation details, I expect results won't be reproducible without significant investment, if at all. However, ideas are easily understood and can be built upon.

Response:

We have added the Code availability section in the revised paper. Specifically, the highway traffic simulator and the autonomous vehicle simulation platform are publicly available, as described in the text and the relevant references[5, 6]. The codes used for the generation of naturalistic and adversarial driving environment are available from the corresponding author on reasonable request. Moreover, we have also added the simulation architecture and algorithm flowchart of our approach in the Supplementary Materials in the revised paper. Please see our Response to the fifth question for details.

8. I should emphasis that all experiments and evaluations are in simulation and no real world tests have been conducted of any kind.

Response:

It is true that all experiments and evaluations are conducted in simulation in this paper. The major difficulty to apply our approach in an AV test track is to control the

[5] A. Dosovitskiy, G. Ros, F. Codevilla, A. Lopez, V. Koltun, CARLA: An open urban driving simulator. https://arxiv.org/abs/1711.03938 (2017).

[6] https://github.com/eleurent/highway-env.

background vehicles as in NADE. Towards addressing this difficulty, in our previous work[7], we developed an augmented reality testing platform, which combines a closed AV test track and a microscopic simulation model, to generate virtual background traffic and send the virtual vehicle information to the autonomous vehicle in the test track using wireless communications. Based on this platform, we tested a level 4 Lincoln MKZ hybrid AV at the Mcity test track of the University of Michigan using the scenario generation method we developed in our previous work. How to utilize the augmented reality testing platform for the implementation of the NADE is interesting, and we leave that for future work.

Overall, the paper presents and original and thought provoking approach. While I suspect in its current form it might have very limited utility for the reasons mentioned above, I feel optimistic that it can seed some great future work in this important problem space. Given this, I would recommend Weak Accept for this paper.

Response:

Thanks for your positive feedback! We agree that further development and enhancement are required for improving the practical utility of our approach. We are working on this direction and will report our results in the future.

[7] Feng, S., Feng, Y., Yan, X., Shen, S., Xu, S. and Liu, H.X., 2020. Safety assessment of highly automated driving systems in test tracks: A new framework. Accident Analysis & Prevention. DOI: 10.1016/j.aap.2020.105664.

Reviewer 3

The paper presents a paradigm for testing AI-powered autonomous vehicles (AV) using adversarial examples for the driving behaviors of the background vehicles (BV) in a highway driving scenario.

The initial behaviors of the background vehicles are first modeled from real-world highway scenes using standard object and lane detection techniques. In a subsequent step, these are re-modeled using an adversarial examples based on surrogate (I guess deep learning) models.

My main concerns with this paper are:

- lack of novelty. The methodology described in the paper has been already presented in papers describing adversarial networks or modeling of the driving scene's dynamics (e.g. [1]).

[1] Mikael Henaff, Alfredo Canziani, Yann LeCun, MODEL-PREDICTIVE POLICY LEARNING WITH UNCERTAINTY REGULARIZATION FOR DRIVING IN DENSE TRAFFIC, ICRL 2019.

Response:

Most existing generative adversarial networks or modeling of the driving dynamics (e.g., [1]) focus on the training of autonomous vehicles (AVs), and cannot be directly applied for the testing and evaluation of AVs. We note that, on a highway driving scenario, all AV models developed in [1] have failure rates (collisions or drive off the road) larger than 20%, which are much worse than the performance of human-driven vehicles (see Fig. 6a in [1]). This might be OK during the training process, the results, however, cannot be used for driving intelligence testing of autonomous vehicle. In this paper, we focus on the testing problem of AVs, which is also challenging. It has been argued[1] AVs would have to be driven hundreds of millions of miles and sometimes hundreds of billions of miles in naturalistic driving environment (e.g., the dynamics model in [1]) to demonstrate their safety at the level of human-driven vehicles, because of the rareness of safety-critical events. It is inefficient even under aggressive simulation schemes.

Moreover, the learning process of background vehicles is not to adversarially defeat the AV agent, which is usually the case for existing adversarial networks, but to test the AV agent in a naturalistic and adversarial manner. In this way, the testing results (such as accident rates of different accident types) of AVs in the generated environment

[1] Kalra, N. and Paddock, S.M., 2016. Driving to safety: How many miles of driving would it take to demonstrate autonomous vehicle reliability? Transportation Research Part A: Policy and Practice, 94, pp.182-193.

can be unbiased, as opposed to only finding worse cases, and the testing time can be significantly reduced to address the inefficiency issue.

To make the contribution of the paper clear, we have rewritten the Introduction section. In the revised paper, we have highlighted the importance and challenges of the AV testing problem, discussed the limitations of state-of-the-art approaches, and summarized the contributions of our study. Specifically, our approach to the construction of a simulation or test-track based AV testing environment has the following three contributions: First, our approach generates driving environment that provides spatiotemporally continuous testing scenarios for AVs, which cannot be handled by existing scenario-based approaches. Suppose you want to test an AV in an urban environment, our approach can drive the AV continuously for miles during one test, interacting with multiple background vehicles and experiencing different adversarial maneuvers. Second, the generated environment provides statistically accurate testing results. Our approach ensures that the testing results (such as accident rates of different accident types) of AVs in the generated environment are unbiased with the NDE. Third, the generated environment addresses the inefficiency issue of the NDE. Comparing with the NDE, our approach reduces the testing time with multiple orders of magnitude for the same evaluation accuracy. Please see the Introduction section in the revised paper.

[1] Mikael Henaff, Alfredo Canziani, Yann LeCun, MODEL-PREDICTIVE POLICY LEARNING WITH UNCERTAINTY REGULARIZATION FOR DRIVING IN DENSE TRAFFIC, ICRL 2019.

- the driving model is overly simplistic. The main challenge with AVs today is driving in complex and cluttered environments, such as cities or crowded streets. However, the paper presents just a simulated highway driving, without even considering different actors (all BVs are the same; modeling trucks or motorcycles would help). Autonomous driving is mostly solved for the case of highway driving.

Response:

We agree that the case study in this paper has several simplifications for the convenience of experiments. However, the theoretical analysis and methodological framework are not limited to these simplifications. As shown in the Methods section, our analysis is generic for the agent-environment framework with general environment (x), action space (u), and critical variables (x_c). Therefore, our approach can also be applied to more complex scenarios such as city driving, and different road users such as trucks and motorcycles can also be considered, as long as sufficient naturalistic driving data is available. We have also added more discussions on the data requirement of our approach. Please see the Discussion section in the revised paper.

- the authors mention that a lane change is detected based on a threshold distance from the ego-vehicle to the lane markings (see Fig. 2). This is quite a poor choice, since it may not be applicable at all to the actual dynamics of a lane change. Typically some form of temporal dynamics learning should be involved.

Response:

In this paper, for the identification of lane-change maneuvers, we have considered temporal dynamics when analyzing lateral distances of ego vehicles to lane markings. First, before the identification step, all trajectories have been filtered to guarantee the temporal continuity, removing abnormal data points (e.g., distances to other vehicles change abnormally). Second, a lane-change maneuver is identified mainly according to the temporal change of the lateral distances from the vehicle center to the lane marking. At the moment the vehicle made the lane change (crossing the lane marking), the lateral distances to both the lane markings will change substantially, as shown in the figures below. Third, after a lane-change maneuver is identified, we trace backward to find the moment when the lane change starts, where the temporal changes of the lateral distances are also analyzed. Similar techniques are also utilized by previous studies[2].

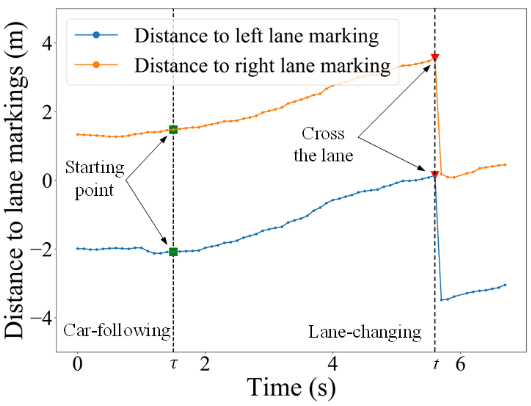

Vehicle is doing a left lane change.

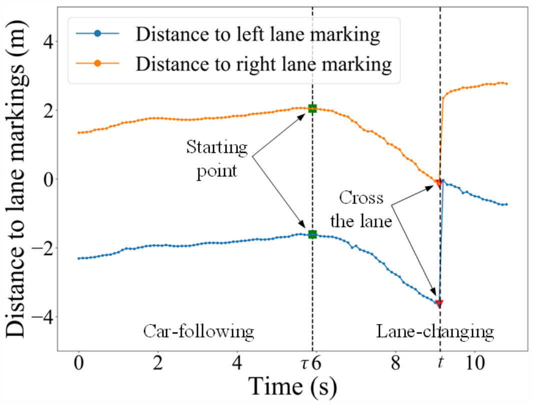

Vehicle is doing a right lane change.

- in a paper describing testing of autonomous vehicles, there is not a single paragraph mentioning how the proposed system maps to functional safety standards such as the ISO 26262 or the ASIL levels.

---

[2] de Gelder, E., Manders, J., Grappiolo, C., Paardekooper, J.P., Camp, O.O.D. and De Schutter, B., 2020. Real-World Scenario Mining for the Assessment of Automated Vehicles. In 2020 23st International Conference on Intelligent Transportation Systems (ITSC), IEEE. The preprint can be found in https://arxiv.org/abs/2006.00483.

Response:

We have rewritten the Introduction section to better motivate the paper and highlight the contributions. Current testing procedures for human-driven vehicles, such as Federal Motor Vehicle Safety Standards (FMVSS)[3] and ISO 26262, only regulate automobile safety-related components, systems, and design features, without consideration of driving intelligence in completing driving tasks. To fill this gap, new testing methods or standards are being discussed by several professional groups or research organizations, such as SAE On-road Automated Driving Technical Committee, IEEE P2846[4], UL 4600 Standard[5], ISO 21448[6], and PEGASUS research project[7], some of which the authors are also involved in. For example, ISO 21448 proposes the definition of SOTIF (Safety of the Intended Functionality) to consider the safety of AVs in situations without a system failure, towards filling the gap of ISO 26262 that defines the Automotive Safety Integrity Level (ASIL) levels. To the best of the authors' knowledge, to date there is no consensus nor standard procedures on how to test and evaluate AVs. During the past few years, although the problem of AV testing has been investigated extensively by people from multidisciplinary research domains such as mechanical engineering, software engineering, and artificial intelligence (AI), the theory and methods to support such testing and evaluation are lacking. We have rewritten the Introduction section to better motivate the paper and highlight the contributions. Please see the Introduction section in the revised paper.

- simulated environments tend to be highly biased towards their real-world counterparts. The paper presents a theoretical proof that the proposed system is unbiased. However, this claim is not proven in any real-world experiments.

Response:

It is true that all experiments and evaluations are conducted in simulation in this paper. The major difficulty to apply our approach in an AV test track is to control the background vehicles as in NADE. Towards addressing this difficulty, in our previous work[8], we developed an augmented reality testing platform, which combines a closed AV test track and a microscopic simulation model, to generate virtual background traffic and send the virtual vehicle information to the autonomous vehicle in the test track

---

[3] https://www.nhtsa.gov/laws-regulations/fmvss

[4] https://sagroups.ieee.org/2846/

[5] https://edge-case-research.com/ul4600/

[6] https://www.iso.org/standard/70939.html

[7] https://www.pegasusprojekt.de/en/about-PEGASUS

[8] Feng, S., Feng, Y., Yan, X., Shen, S., Xu, S. and Liu, H.X., 2020. Safety assessment of highly automated driving systems in test tracks: A new framework. Accident Analysis & Prevention. DOI: 10.1016/j.aap.2020.105664.

using wireless communications. Based on this platform, we tested a level 4 Lincoln MKZ hybrid AV at the Mcity test track of the University of Michigan using the scenario generation method we developed in our previous work. How to utilize the augmented reality testing platform for the implementation of the NADE is interesting, and we leave that for future work.

- in this reviewer's opinion, the key to self-driving cars (both implementation and testing) lies in overcoming the data bottleneck. We simply cannot expect to train these systems solely by throwing large quantities of data to them.

Response:

We completely agree with the reviewer that we cannot train the AV agents by simply throwing large quantities of data to them. This paper proposes one way to use the large quantity of naturalistic driving data effectively. One key idea of our approach is not to reproduce testing scenarios solely by throwing the NDD (i.e., NDE) to the AV under test, but to generate the NADE leveraging the knowledge of AVs. Our theoretical analysis and methodological framework provide the foundation for achieving this goal.

We also agree that our approach does require large amount naturalistic driving data to model the driving behaviors of background vehicles in NDE. Relative position and speed information of the ego vehicle and surrounding vehicles are needed to construct the empirical distributions of vehicle interaction behaviors. For complex driving environment, millions of data points would be required to represent the variability of the environment. Fortunately, with the deployment of vehicle-based and infrastructure-based perception sensors, nowadays the data can be collected with lower cost and become more accessible[9]. We have revised the paper accordingly. Please see the Discussion section in the revised paper.

[9] Wang, W., Liu, C. and Zhao, D., 2017. How much data are enough? A statistical approach with case study on longitudinal driving behavior. IEEE Transactions on Intelligent Vehicles, 2(2), pp.85-98.

Reviewer #1 (Remarks to the Author):

The paper offers a positive contribution to Autonomous Vehicle research and literature set. I found it challenging as I didn't agree with many of the key parts of the first draft. That said, the quality and insights were developed and informed. You could easily appreciate the work and investment put into the research. The changes and response were insightful and certainly added further value to the research. It provides an excellent resource to autonomous vehicle research in this specific context and also in the wider domain. In light of the comprehensive and well founded responses to my review and the other reviewers, I can confirm the paper is of a high quality in terms of research and communication. Accordingly, I recommend the should now be published in this journal.

Dr Martin Cunneen

Reviewer #3 (Remarks to the Author):

The authors addressed the comments and questions posed by the reviewers. Appreciate the rewriting of the sections to better address the challenges.

As mentioned also by reviewer 1, NADE seems not to be a magic bullet for AV development. Hopefully in your follow-up work you could address more complicated scenarios (including real-world deployments), with a more diverse population of traffic participants.

As a conclusion, the paper has been improved from the original version. I feel confided that the authors will better link real-world AV development and testing to their NADE framework in the future. I recommend Accept for the paper.

Sorin Grigorescu
www.rovislab.com

Response to the Reviewers' Comments

Reviewer 1

The paper offers a positive contribution to Autonomous Vehicle research and literature set. I found it challenging as I didn't agree with many of the key parts of the first draft. That said, the quality and insights were developed and informed. You could easily appreciate the work and investment put into the research. The changes and response were insightful and certainly added further value to the research. It provides an excellent resource to autonomous vehicle research in this specific context and also in the wider domain. In light of the comprehensive and well founded responses to my review and the other reviewers, I can confirm the paper is of a high quality in terms of research and communication. Accordingly, I recommend the should now be published in this journal.

Response:

We thank the reviewer for the positive evaluation and constructive suggestions of the work.

Reviewer 3

The authors addressed the comments and questions posed by the reviewers. Appreciate the rewriting of the sections to better address the challenges.

As mentioned also by reviewer 1, NADE seems not to be a magic bullet for AV development. Hopefully in your follow-up work you could address more complicated scenarios (including real-world deployments), with a more diverse population of traffic participants.

As a conclusion, the paper has been improved from the original version. I feel confided that the authors will better link real-world AV development and testing to their NADE framework in the future. I recommend Accept for the paper.

Response:

We thank the reviewer for the positive evaluation and constructive suggestions of the work. We will investigate how to utilize the NADE framework for the real-world AV testing in our future work.
